# Macromolecular Insights into the Altered Mechanical Deformation Mechanisms of Non-Polyolefin Contaminated Polyolefins

**DOI:** 10.3390/polym14020239

**Published:** 2022-01-07

**Authors:** Ruben Demets, Marie Grodent, Karen Van Kets, Steven De Meester, Kim Ragaert

**Affiliations:** 1 Circular Plastics, Department of Circular Chemical Engineering, Faculty of Science and Engineering, Maastricht University, Urmonderbaan 22, 6162 AL Geleen, The Netherlands; ruben.demets@ugent.be; 2 Laboratory for Circular Process Engineering (LCPE), Department of Green Chemistry and Technology, Faculty of Bioscience Engineering, Ghent University, Campus Kortrijk, Sint-Martens-Latemlaan 2B/5, 8500 Kortrijk, Belgium; karen.vankets@ugent.be (K.V.K.); steven.demeester@ugent.be (S.D.M.); 3 Dutch Polymer Institute (DPI), 5600 AX Eindhoven, The Netherlands; 4 Centre for Polymer and Material Technologies (CPMT), Department of Materials, Textiles and Chemical Engineering, Faculty of Engineering and Architecture, Ghent University, Technologiepark 130, 9052 Ghent, Belgium; marie.grodent@hotmail.com

**Keywords:** immiscible polymer blends, polyolefins, deformation mechanisms, commodity plastics, mechanical recycling, structure–property relationships

## Abstract

Current recycling technologies rarely achieve 100% pure plastic fractions from a single polymer type. Often, sorted bales marked as containing a single polymer type in fact contain small amounts of other polymers as contaminants. Inevitably, this will affect the properties of the recycled plastic. This work focuses on understanding the changes in tensile deformation mechanism and the related mechanical properties of the four dominant types of polyolefin (PO) (linear low-density polyethylene (LLDPE), low-density polyethylene (LDPE), high-density polyethylene (HDPE), and polypropylene (PP)), contaminated with three different non-polyolefin (NPO) polymers (polyamide-6 (PA-6), polyethylene terephthalate (PET), and polystyrene (PS)). Under the locally elevated stress state induced by the NPO phase, the weak interfacial adhesion typically provokes decohesion. The resulting microvoids, in turn, initiate shear yielding of the PO matrix. LLDPE, due to the linear structure and intercrystalline links, is well able to maintain high ductility when contaminated. LDPE shows deformation similar to the pure material, but with decreasing ductility as the amount of NPO increases. Addition of 20 wt% PA-6, PET, and PS causes a drop in strain at break of 79%, 63%, and 84%, respectively. The typical ductile necking of the high-crystalline HDPE and PP is strongly disturbed by the NPO phase, with a transition even to full brittle failure at high NPO concentration.

## 1. Introduction

Plastic recycling is dominated by packaging waste streams. Typical polymers in this packaging sector include polyamide (PA), polyethylene terephthalate (PET), polystyrene (PS), polypropylene (PP), and polyethylene (PE), which, in turn, is subdivided into linear low-density polyethylene (LLDPE), low-density polyethylene (LDPE), and high-density polyethylene (HDPE). PP and PE belong to the family of polyolefins (POs), while PA, PET, and PS do not. For the sake of this manuscript, they will be called non-polyolefins (NPOs). Single-use packaging often has a short lifetime, thus contributing to a large amount of waste. Although recycling systems are quickly developing and sorting technologies improve each day, for some products, e.g., multi-layered films, trays, etc., it is very difficult to separate the different polymers, resulting in the cross-contamination of one polymer by another. Therefore, these commingled waste stream products often end up being incinerated at best for energy recovery [1,2].

The multiple layers in flexible packaging consist of various polymer layers to combine their different properties (barrier properties, seal ability, chemical resistance, etc.) [3,4]. Roughly estimated from the literature, the amount of flexible multilayer packaging in Europe would be approximating 1.044 million tons per year (2019), but this is expected to be an underestimation by more than double [5,6]. Multi-layered packaging trays are another example of a product that is composed of various polymers to combine their different properties. Usually, they are produced from PE, PP, PS, and/or PET [7,8]. In addition—for example, in the furniture market—different polymers are combined to optimize the functionality of the product. For example, carpets are often produced from a PP carpet backing and PA or PET yarns [9,10]. As the separation efficiency of these polymers is low, different POs and NPOs will have to be recycled together as a blend.

A polymer blend is a system consisting of two or more mechanically mixed polymers, and each polymer preserves its original properties regardless of the blend composition [11]. A simple prediction of the blend properties by making a summation of properties is not as straightforward as expected, as most polymers mentioned above give way to immiscible, phase-separated morphologies. POs such as HDPE, LLDPE, LDPE, and PP are inherently immiscible with NPOs such as PA-6, PET, and PS [12,13]. This results from a lack of interaction between the polymer phases originating from the chemical nature of the polymers, the blend’s composition, and the surrounding temperature. However, there is also a strong effect of the processing temperature and shear rate, and both affect the viscosity to volume fraction ratio. The latter will control how the viscoelastic phase separation mechanism will occur during processing and, hence, contributes to the final morphology of the blend [14].

The morphology of a blend will be influenced by the composition of the blend and, thereby, take part in the particle size and possible continuity of the minority phase. If a blend has a composition in which one polymer dominates by volume fraction and/or by its low viscosity, it can act as a continuous phase (matrix) separating the other polymer(s) into distinguishable particles. The processing conditions largely affect the final blend morphology and may give rise to a droplet-shaped, a fibrillated, a laminar, and/or a co-continuous morphology [1,11,15,16,17,18]. Which of these morphologies will dominate depends on the flow behavior during processing and the shear stresses, which play a part in the breakup or coalescence of the dispersed phase. Moreover, the importance of the flow behavior increases as the flow behavior of each polymer compared to the other becomes more asymmetric [19,20]. 

The final morphology will largely determine the deformation behavior and the resulting mechanical properties. The morphology of a polymer blend can be described in two ways: (i) the microscopic structure of the phase-separated polymers, which is mainly defined by the viscosity to volume fraction ratio, and (ii) the crystalline morphology of the individual blend polymers [21]. Polymers can be semi-crystalline with a high tendency to crystallization (HDPE, PP, PET, PA) or a low tendency to crystallization (LDPE, LLDPE), or they can be amorphous (PS) [22,23]. In addition, the deformation mechanisms occurring in immiscible polymer blends also depend on the interfacial adhesion and thus the degree of stress transfer between the different polymer phases [21].

Three deformation regions can be defined for a mono polymer: (i) the elastic region, (ii) the combined elastic–plastic region, and (iii) the plastic region. These regions of deformation can also be distinguished in blend materials [24,25]. However, the deformation behavior of a polymer blend is complicated by the fact that the minor phase can act as a rubber particle, reinforce the matrix, or have no particular influence due to its size and shape. Depending on the microstructure, the failure of the blend can occur in either of the three deformation regions and correlates with the critical yield stress. 

The following failure mechanisms during elongation can occur in decreasing order of strain: shear yielding (in the crystalline phase) and shear banding (in the amorphous phase), crazing (in the amorphous phase), cavitation (in the amorphous phase), and cracking (in both phases). For both crazing and cavitation, a measurable volume increase can be noticed due to (micro)void formation [26,27,28]. The deformation of an immiscible binary polymer blend depends on three dominating factors: (i) the particle size of the dispersed phase and its dispersion in the matrix, (ii) the differences in the individual elastic moduli of each polymer in the blend, and (iii) the crystallinity of the blend polymers. If the dispersed particles in the polymer blend are droplet-shaped, have diameters smaller than 1 µm, and are well-dispersed, then the matrix will independently carry most of the uniaxial load, even after yielding of the minor phase with a higher modulus [29,30]. Immiscible polymer blends tend to deform on a microscopic level by (micro)cavitation or crazing caused by decohesion at the interphase, shear banding or shear yielding of the matrix initiated on the minor phase particles, and cracking throughout the matrix around or across the minor phase particles (depending on the aspect ratios of the particles) [30,31,32].

In the above-mentioned deformation mechanisms, a strong, dominating aspect is the resistance to deformation of the crystalline phase. PO semi-crystalline polymers have a glass transition temperature that is lower than room temperature. Thus, they will have crystalline domains that are interconnected by tie molecules and entanglements of the amorphous phase [33]. Both the semi-crystalline and the amorphous phases will absorb the deformation energy, starting with the elastic stretching of the amorphous regions. With increasing strain, crystal deformation triggers minor plastic deformation. This is due to isolated inter- and intra-lamellar slips and rotational slips. By further increasing the strain, the amount of plastic deformation increases until the yield point is reached. This point coincides with the maximum engineering stress before necking and is attributed to the collective inter- and intra-lamellar slips, such as separation into smaller lamellae blocks and rearrangements in the amorphous phase. By further increasing the strain after the maximum stress is reached, coarse slip of the crystal blocks occurs to align them in the tensile direction. At a higher strain, fibrillation of the crystal blocks can be initiated. Finally, disentanglement of the highly elongated polymer chains occurs and causes strain hardening [24,34,35,36].

Previous research examined the altered properties and underlying deformation mechanisms of PO/PO blends [23]. The focus of this research is on the altered deformation mechanisms and mechanical properties of PO matrices contaminated with NPO plastics. For the sake of completeness, the influences on the mechanical properties of PO contaminations in the same NPO plastics were also tested. Twelve different binary blend series of PO with NPO were produced and tested over the whole composition range (0, 5, 10, 20, 50, 80, 90, 95, and 100 wt%). The plastics used are relevant for the blend structures that can be encountered in industrial recycling processes. Four PO (LDPE, LLDPE, HDPE, and PP) and three NPO (PA-6, PET, and PS) were used for this purpose. The importance of understanding the impact of the contamination of one polymer on a matrix of another polymer is of high relevance in the proper assessment of the quality of recycled plastics [37].

## 2. Materials and Methods

### 2.1. Materials

Six commercial polymer grades were used in this work. Four POs were used: LLDPE (an ethylene 1-hexene copolymer), LDPE, HDPE, and PP. These PO materials were each combined with one of the three NPO materials used: PET, PA-6, and PS. More information on each grade can be found in Table 1.

### 2.2. Sample Preparation

In this research, twelve binary PO/NPO blend series were produced with the polymers listed in Table 1. Each of the NPO polymers was combined with each of the PO materials, producing twelve blends covering the entire composition range (0–5–10–20–50 wt% NPO in PO, and vice versa). The polymer pellets were physically blended by hand mixing rather than compounded, to better represent the materials encountered in industrial mechanical recycling processes.

ISO 527-2/1A test bars were produced by injection molding using an Engel 28-ton injection machine (Engel e-victory, Schwertberg, Austria). Temperature profiles were chosen according to the highest melting polymer. This ensured that both polymers were fully melted. All temperature profiles applied, with temperatures from hopper to nozzle, are depicted in Table 2. The mold temperature was kept constant at 40 °C for all blends.

### 2.3. Mechanical Characterization

Tensile tests were conducted according to ISO 527-2 on an Instron 5565 tensile testing machine (Norwood, MA, USA) equipped with a 5 kN load cell. The test bars were conditioned for at least five days, after which the tensile tests were executed in the same conditioned laboratory (23 ± 2 °C, 50 ± 10% relative humidity). For the precise determination of the elastic deformation, an Instron clip-on extensometer (Instron type 2630-100—gauge length 50 mm) was used. A pre-load of 40 N was set for all samples. The elastic modulus (E) was determined as a secant between 0.05% and 0.25% engineering strain. A crosshead speed of 1 mm/min was maintained up to 0.3% engineering strain. After removal of the extensometer, the crosshead rate was raised to 50 mm/min and testing proceeded until sample fracture. Yield strength (σ_y_) and strain at yield (ε_y_) were determined at zero slope for most materials. For the blends with a majority of LDPE and LLDPE, however, these properties were determined by the 0.2% offset method, as these materials lack a zero-slope plateau. Tensile testing also provided values for strain at break (ε_b_). The reported average values and standard errors are based on 10 tensile tests.

### 2.4. Differential Scanning Calorimetry (DSC)

To determine the degree of crystallinity (Χ_c_) of the blend components, DSC measurements were executed on a Netzsch DSC 214 Polyma (Selb, Germany). Approximately 10 mg of sample mass was taken from the middle of the tensile test bar. Per test, two heating/cooling cycles under nitrogen atmosphere were performed from 30 °C up to 300 °C. A heating or cooling rate of 10 K/min was set and an isothermal of 5 min was introduced between each heating and cooling run. Χ_c_ was calculated from the first heating cycle according to Equation (1), considering the melting enthalpies (Δ*H_m_*), weight fraction *x* of the polymer in the blend (wt%), and eventual cold crystallization (Δ*H_cc_*). The melting enthalpies of the 100% crystalline polymers ($\Delta H_{m}^{0}$) are as follows: PET 140 J/g, PA-6 230 J/g, PE 293 J/g, PP 207 J/g [38]. All reported values are the average of at least 2 measurements.

(1) $${Χ}_{c}\;\left(\%\right)=\frac{\Delta H_{m}-\Delta H_{cc}}{x\cdot \Delta H_{m}^{0}}\cdot 100\%$$

### 2.5. Scanning Electron Microscopy (SEM)

SEM was used to examine the morphology of the actual deformed samples. SEM micrographs were made on a Phenom Pro (Eindhoven, The Netherlands) with an accelerating voltage of 15 kV. Before SEM analyses, the surfaces of the broken test specimen were sputtered with a gold coating (PLASMATOOL-SC (Wetzlar, Germany)). The SEM images of brittle fractured samples were taken in the longitudinal direction (LD). Higher elongated samples were examined in the transversal direction (TD). On the SEM images shown, a red arrow indicates the direction of the polymer flow.

## 3. Results

In this section, the observed trends in crystallinity and mechanical properties are discussed, as well as the visual macroscopic deformation of the tested tensile specimen of each blend. The subsequent Discussion section analyzes the observed trends and explains the changes in deformation on a macromolecular basis.

### 3.1. Crystallinity

#### 3.1.1. Crystallinity of Mono Materials

Table 3 reports the crystallinity values of the mono materials. The different blend combinations were, each time, processed at the processing temperature of the highest melting polymer; hence, different values per polymer type are reported in Table 3. Neat PET and PA-6 have a crystallinity of 24.9% and 27.4%, respectively. PS is a completely amorphous material and thus has a degree of crystallinity of 0%. Regarding the PO polymers, LLDPE (28.0% to 29.0%) and LDPE (31.9% to 34.2%) have a relatively low degree of crystallinity compared to HDPE (64.1% to 65.6%) and PP (45.2% to 47.8%), which is due to the branched structures of the L(L)DPE polymers [39]. The differences in crystallinity for a neat polymer processed at different temperatures can be attributed to the stronger quenching effect due to an increasing gradient between mold and processing temperature. A complete blend series of a combination of two polymers was always produced on the same day. However, the time between the different series may vary considerably. The notable variations for HDPE and PP may reflect changing ambient conditions on the processing day. It is therefore important to only compare crystallinity values within a single blend series, which were all produced on the same day.

#### 3.1.2. Crystallinity of the Blend Series

**Figure 1:**
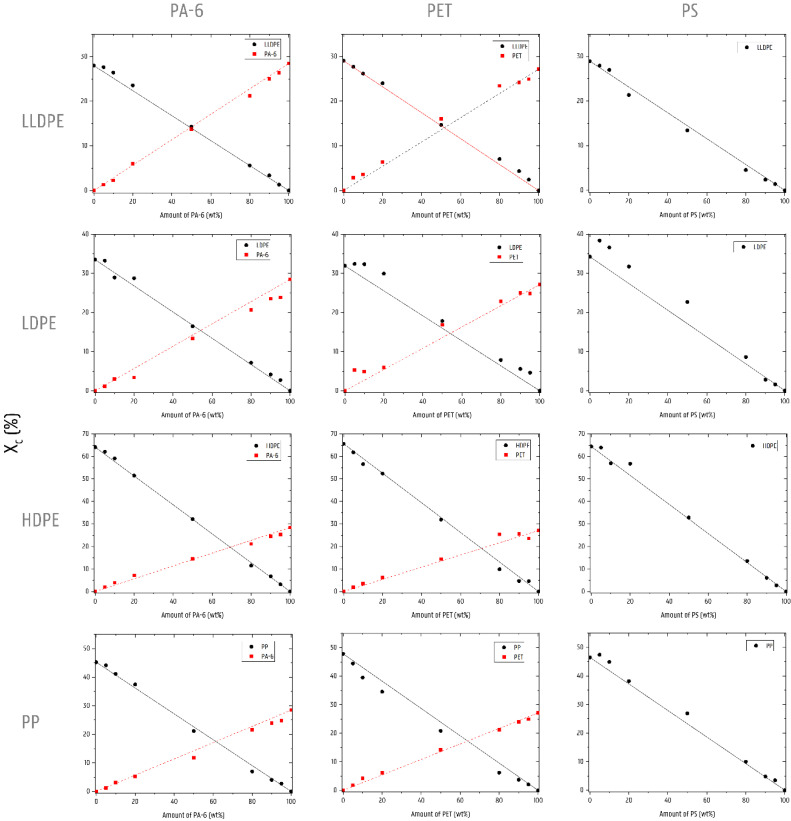
Crystallinity of all blend series. Experimental measured values are represented by symbols (●: PO materials and ■: NPO materials). Lines are used to represent the prediction based on the additivity rule (___: prediction for PO materials and _ _ _: prediction of NPO materials).

Figure 1 gives an overview of the calculated crystallinities measured via DSC. This figure shows the measured data point for each polymer present in the respective blend, as well as the lines representing the prediction for the theoretically expected crystallinity, based on a proportional mixing rule using the crystallinities of the respective mono materials. This supposes an idealized condition wherein the two components crystallize wholly independently of one another. Two irregularities may occur, however, in some of the blend DSC signals, which can influence the calculated values for crystallinity. Firstly, the glass transition of PS (±100 °C) disturbs the signal of the melting peak of the PO in the blends with high amounts of PS (50–95 wt%). Secondly, the occurrence of cold crystallization in the blends containing 50–95 wt% PET interferes with the melting enthalpy value of the polyethylene in PET/PE blends.

**Figure 2:**
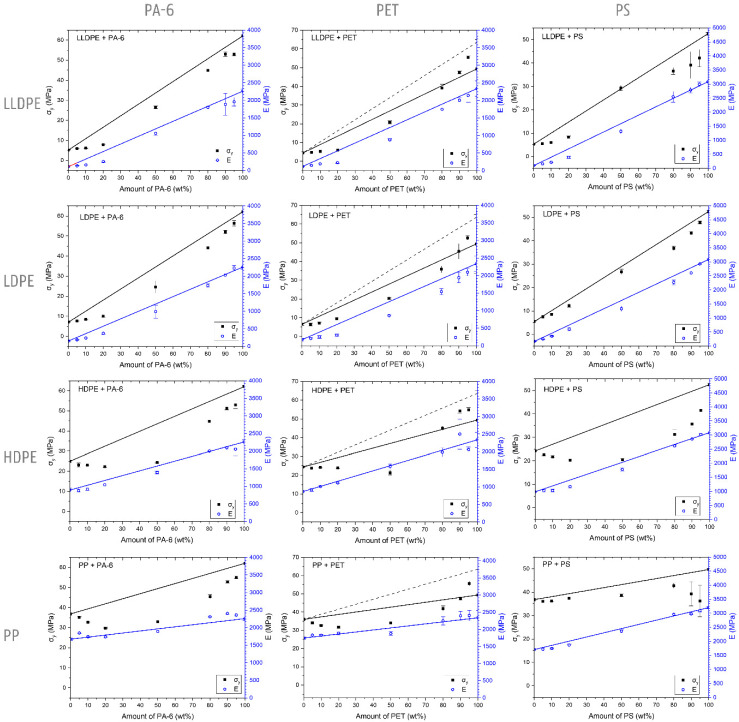
Overview of the σy (■) and E (○) values for the tested blend series. Lines present the prediction based on the additivity rule for σy (black line) and E (blue line). The dashed line (PET blends) presents the predictions for σy for a PET value of 63.6 MPa (average of PET with ε_b_ of minimum 4%).

The addition of PA-6 to an LDPE or LLDPE matrix has a positive effect on the crystallinity of these matrix polymers (with exception of the 10 wt% LDPE/PA-6 blend). HDPE and PP polymers contaminated with PA-6 follow the trend given by the shown additivity rule (Figure 2). The crystallinity of the minority PA-6 phase is in line with additivity, with exception of the 20 wt% LDPE/PA-6, which shows a clear drop in crystallinity. When PA-6 constitutes the matrix phase, a negative effect on the crystallinity upon addition of a PO is observed. This negative impact is especially pronounced for the addition of LDPE. The crystallinity of the PO droplet phase generally follows the additivity rule. However, a decrease has been noted for the 20/80 wt% HDPE/PA-6 and PP/PA-6 blends. On the other hand, an increase compared to the prediction is found for the LDPE/PA-6 materials containing 90 wt% and 95 wt% PA-6.

A significantly higher Χ_c_ was measured for the LDPE matrices to which PET was added. The PET phase itself also has markedly higher crystallinity. For the crystallinities of the LLDPE and HDPE (with exception of the 10 wt% PET blend) matrices contaminated with PET, the additivity trend is followed. PET has a negative influence on the Χ_c_ of a PP matrix. The crystallinity of PET present in an LLDPE, HDPE, or PP matrix is higher compared to the crystallinity of the PET mono material. The crystallinity of the materials in which PET forms the matrix is difficult to evaluate due to the anomaly caused by the cold crystallization peak. For the PP-contaminated materials, the PET matrix crystallinity does follow the proportional rule. Χ_c_ of the PP and HDPE minority phases is negatively impacted. Higher crystallinity is measured for the LDPE and LLDPE droplet phases.

With exception of the 80/20 wt% LLDPE/PS blend and 90/10 wt% HDPE PS blend, a positive effect is observed for the PO matrix materials once PS is added. The effect is particularly strong for the LDPE/PS combinations. The crystallinity of a PO minority phase added to the PS matrix changes little compared to the pure materials. Only the 80/20 wt% combinations of PS/LDPE (increase) and PS/LLDPE (decrease) deviate from the predictions made by additivity.

The respective DSC signals of the different blend materials with PO as the matrix can be found in Figures S1–S4 in the Supplementary Information.

### 3.2. Tensile Deformation and Properties

#### 3.2.1. Mechanical Properties of the Mono Materials

**Figure 3:**
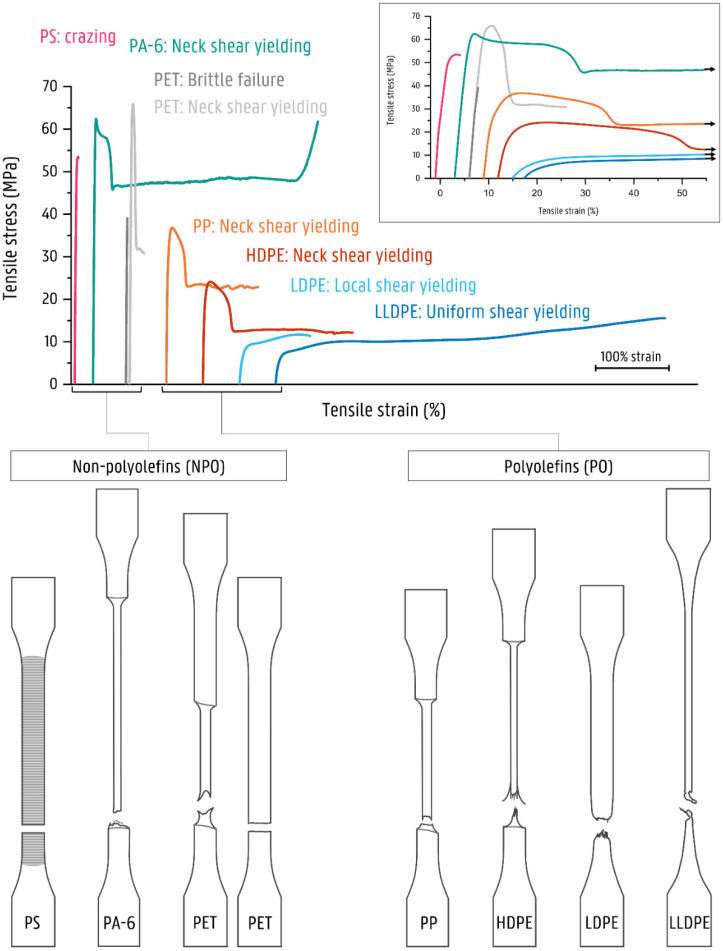
Stress–strain curves for the used mono materials and corresponding deformation mechanism. Crazing is indicated as fine mark lines for the neat PS. The exact values for ε_b_ can be found in Table S1.

**Figure 4:**
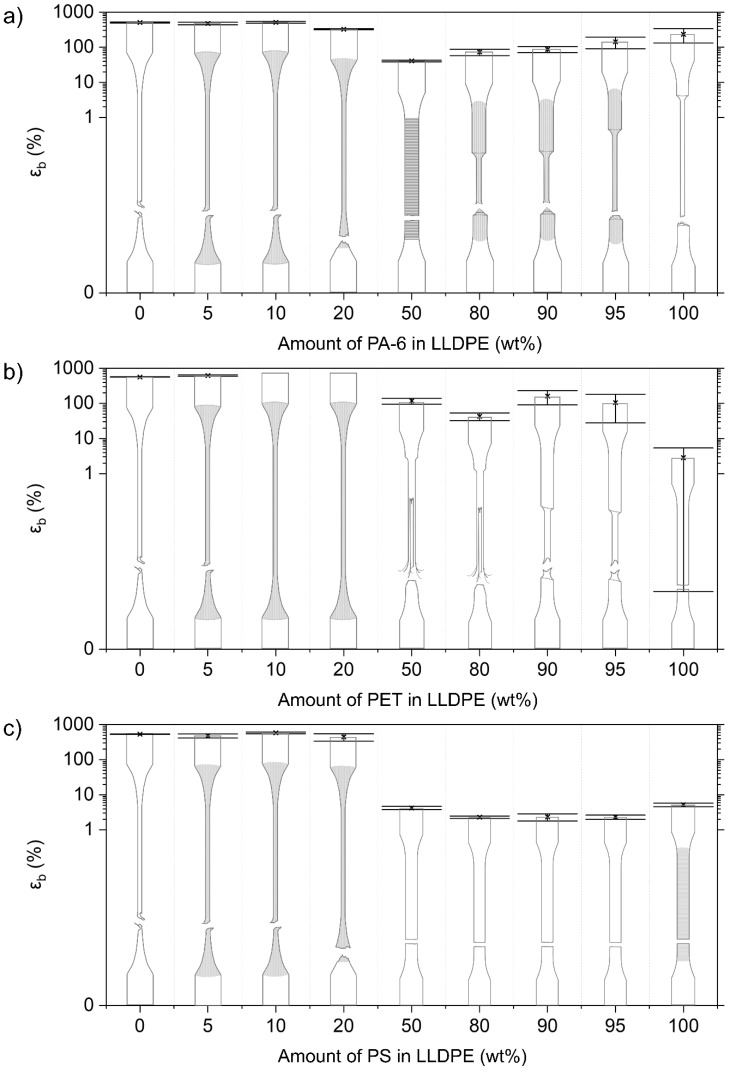
Overview of the deformed samples after tensile testing and respective εb values for the LLDPE blends combined with (**a**) PA-6, (**b**) PET, and (**c**) PS. Crazing is indicated as fine mark lines for the neat PS. Other shading points to stress whitening, which is visible during/after tensile testing. Coarser markings and lines indicate local points of necking initiation.

**Figure 5:**
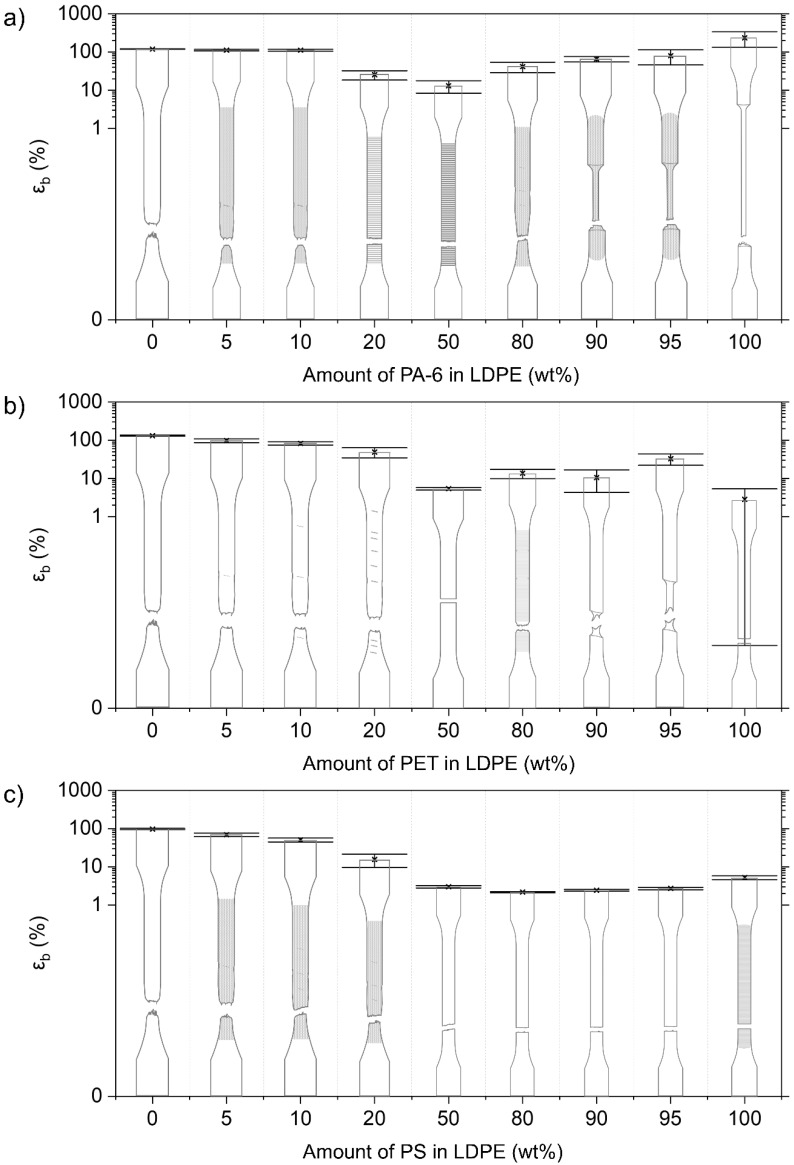
Overview of the deformed samples after tensile testing and respective ε_b_ values for the LDPE blends combined with (**a**) PA-6, (**b**) PET, and (**c**) PS. Crazing is indicated as fine mark lines for the neat PS. Other shading points to stress whitening, which is visible during/after tensile testing. Coarser markings and lines indicate local points of necking initiation.

**Figure 6:**
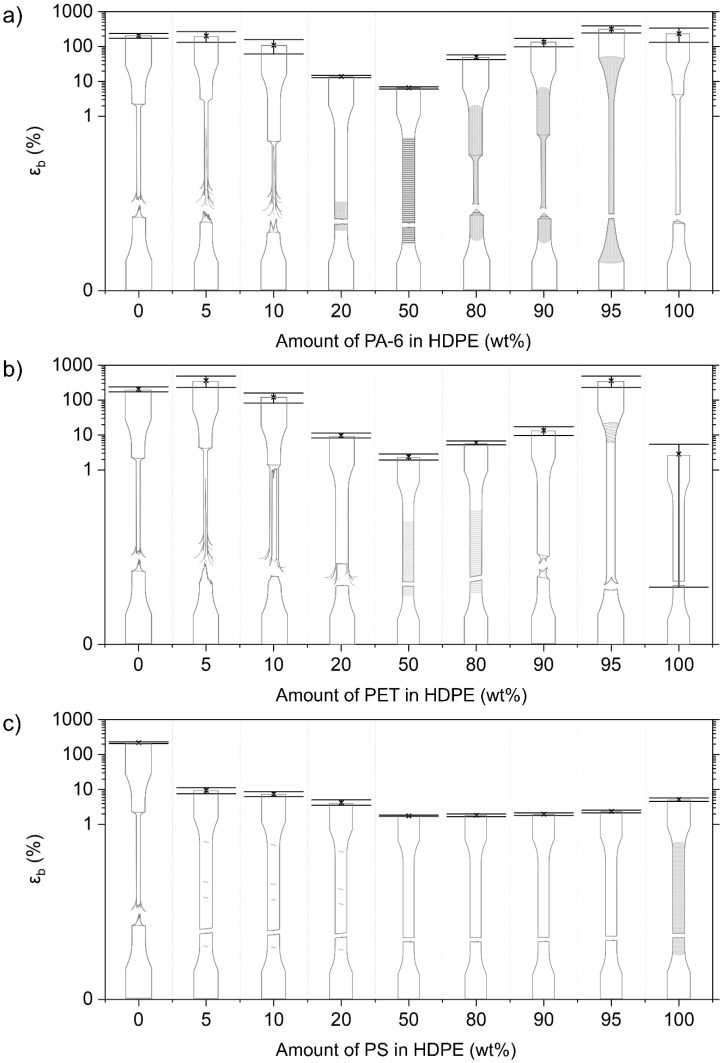
Overview of the deformed samples after tensile testing and respective εb values for the HDPE blends combined with (**a**) PA-6, (**b**) PET, and (**c**) PS. Crazing is indicated as fine mark lines for the neat PS. Other shading points to stress whitening, which is visible during/after tensile testing. Coarser markings and lines indicate local points of necking initiation.

**Figure 7:**
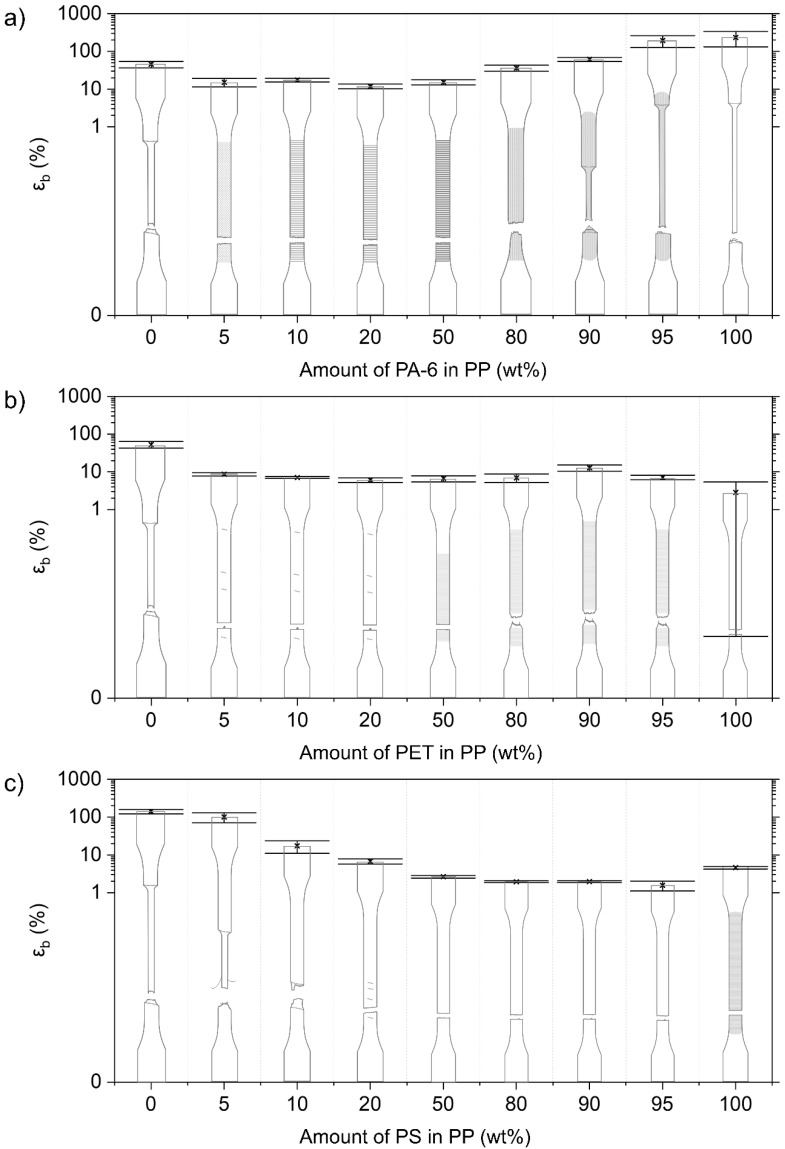
Overview of the deformed samples after tensile testing and respective ε_b_ values for the PP blends combined with (**a**) PA-6, (**b**) PET, and (**c**) PS. Crazing is indicated as fine mark lines for the neat PS. Other shading points to stress whitening, which is visible during/after tensile testing. Coarser markings and lines indicate local points of necking initiation.

The mono materials are each characterized by their specific deformation mechanism, which is reflected in their stress–strain diagrams (Figure 3). The mechanical properties of the used mono materials are included in Table 3. Illustrations of the deformed test specimens are shown in Figure 4, Figure 5, Figure 6 and Figure 7 for the mono materials as well as for each of the tested blends.

Previous research on the deformation mechanisms in PO/PO blends [23] used the same PO grades. As indicated in this previous work [23], HDPE and PP both deform by shear yielding, in which a clear progressive necking is observed. In the deformation of LDPE and LLDPE, this progressive necking phenomenon is lacking. The LDPE shows the initiation of a localized neck. However, necking does not propagate as failure of the sample occurs. The LLDPE material deforms uniformly over the length of the specimen. At higher strains, the LLDPE material also exhibits strain hardening.

The NPO materials are strong in comparison to the POs. PA-6 has a zero slope σ_y_ of 62.1 ± 0.4 MPa. The pure PA-6 material deforms via shear yielding, forming a distinct neck comparable to HDPE and PP. PA-6 has an ε_b_ of 234%, with a rather high standard deviation (102%). However, all tested samples present a ductile deformation with the propagation in the formed neck. The PET material undergoes brittle fracture for most specimens. However, necking is observed for a significant number of test specimens—hence the large variation in the values for σ_y_ (49.4 ± 15.7 MPa) and ε_b_ (2.9 ± 2.5). When only considering the more ductile deformed samples, the PET material has a zero slope σ_y_ of 63.6 ± 2.7 MPa. These discrepancies may be due to the slow crystallization rate of the PET material, causing microstructural differences in crystalline and amorphous regions among the tested samples, which are sufficient to change the local stress situation so that a different deformation mechanism occurs [40,41,42]. Finally, the PS material shows crazing before brittle failure.

#### 3.2.2. Mechanical Properties of LLDPE Blends

##### LLDPE/PA-6

The pure LLDPE material deforms via uniform shear yielding without the occurrence of clear necking (Figure 4a). When adding 5 and 10 wt% PA-6 to the LLDPE matrix, high elongations similar to the pure LLDPE material (ε_b_ = 507 ± 17%) are still attained. The 5 wt% blend has a slightly lower ε_b_, while the material with 10 wt% PA-6 is not significantly different from the pure LLDPE. Upon adding 20 wt% PA-6 contaminant, a clear decrease in ε_b_ is observed (327 ± 12%). For these LLDPE/PA-6 blends, the same uniform deformation as the virgin LLDPE can be observed, but stress whitening is present. E and σ_y_ (0.2% offset method) show a slight increasing trend with rising amounts of PA-6.

When PA-6 is the majority component, clear neck formation is observed for each of the tested blend combinations (5, 10, and 20 wt%). ε_b_ values fall with higher content of LLDPE. σ_y_ (zero slope) is reduced compared to the pure PA-6 material (62.1 ± 0.4 MPa) for the blends with 5 wt% (52.9 ± 0.8 MPa) and 10 wt% (53.0 ± 1.1 MPa) LLDPE. σ_y_ decreases to 44.9 ± 0.5 MPa for the 20 wt% PA-6/LLDPE blend. E values follow the rule of additivity. The 50/50 wt% LLDPE/PA-6 blend shows brittle fracture, yet it still attains an ε_b_ of 41 ± 2%.

##### LLDPE/PET Blends

For the LLDPE blends contaminated with PET, Figure 4b shows the same uniform deformation as for blends with LLDPE as the majority phase. When 5 wt% PET is added, a significant increase in ε_b_ is measured compared to the pure LLDPE (569 ± 7% versus 622 ± 26%). The 10 wt% and 20 wt% LLDPE/PA-6 blends both reach the maximum strain value for the tensile machine, and hence do not fracture during tensile testing. Again, stress whitening is observed in the blend materials. The same slight increase in E and σ_y_ values is observed as in the LLDPE/PA-6 blends.

The pure PET material generally shows a brittle fracture. However, in some of the PET samples, a neck was formed, in which the deformation propagated. For this reason, in Figure 2, two lines representing additivity for σ_y_ are shown for the different blend series with PET. The full black line represents the additivity rule using all tested samples (σ_y_ = 49.4 ± 15.7 MPa), whereas the dashed black line represents only the ductile deformed PET samples (σ_y_ = 63.6 ± 2.7 MPa). For the blends with PET contents of 50 wt% and above, this necking phenomenon is always observed. The ε_b_ values for the 95 wt% and 90 wt% LLDPE in PET mixtures are 106 ± 78% and 162 ± 71%, respectively. Moreover, high standard errors are noted for these samples. Despite the high amount of LLDPE present, the 80/20 wt% (118 ± 22%) and 50/50 wt% (43 ± 11%) LLDPE/PET blends reach relatively high ε_b_ values. However, these high values are a result of the macroscopic fibrillation of the sides of the injection-molded specimens, while the core is already broken. The addition of LLDPE to a PET matrix causes reductions in σ_y_ (Figure 2). E values for these blends are within a 10% error range of additivity.

##### LLDPE/PS Blends

The LLDPE matrices with PS contamination once again show a uniform deformation similar to that of the pure LLDPE polymer. ε_b_ values above 400% are measured for these blends. E and σ_y_ increase slowly. Upon addition of 20 wt% PS, a fourfold increase in E is achieved compared to the virgin LLDPE.

For blends of 50–100 wt% PS, a brittle failure is observed. Noteworthy is the higher ε_b_ for the 50 wt% blend compared to the other blends with PS in excess. In contrast to the pure PS material (which is transparent), no crazing is visible in the blend materials. σ_y_ shows a pronounced decrease with higher content of LLDPE. E values for the 80 wt%, 90 wt%, and 95 wt% PS blends follow the proportional line (Figure 2). 

#### 3.2.3. Mechanical Properties of LDPE Blends

##### LDPE/PA-6 Blends

The 5 wt% and 10 wt% PA-6-contaminated LDPE samples show similar deformation to the pure LDPE material (Figure 5). Only a small decrease in ε_b_ is measured for these blends. These two blend materials do show stress whitening, and marks are apparent on the specimen, showing the onset of a second neck. However, the necking, as with the pure material, does not propagate in a stable neck, and failure occurs at around 112% strain. For the 20 wt% and 50 wt% PA-6 in LDPE blends, brittle fracture is observed instead, and the ε_b_ drops to 25 ± 7% and 13 ± 5%, respectively. As with the above-mentioned blends with LLDPE as the majority polymer, a slight increase in E and σ_y_ is noted, as shown in Figure 2. 

The blends with a PA-6 amount of 90 wt% and 95 wt% show clear neck formation, which is also characteristic for the pure PA-6. However, this necking does not propagate as much as the pure material, resulting in lower ε_b_ values. At a contamination level of 20 wt% LDPE, the material tends to form a neck, but the material fails before this neck can fully propagate. σ_y_ drops significantly with increasing amounts of LDPE. The values of E are within a 10% range of additivity.

##### LDPE/PET Blends

For the LDPE blends contaminated with PET (5 wt%, 10 wt%, and 20 wt%), the deformation mechanism of the pure LDPE is observed. These samples again show marks of potential second neck initiation locations. At higher levels of PET contamination, even more of these spots can be noticed. There is a stronger decrease in ε_b_ for the 5 wt% and 10 wt% PET blends, compared to the LDPE/PA-6 blends. ε_b_ decreases from 132 ± 5% for the pure LDPE to 98 ± 11% (5 wt%), 83 ± 8% (10 wt%), and 49 ± 14% (20 wt%). Figure 2 shows, again, a slight increasing trend for E and σ_y_. The 50 wt% blend shows brittle failure and has an ε_b_ of 5 ± 0%.

The addition of LDPE (5 wt%, 10 wt%, and 20 wt%) to a PET matrix results in necking of the blend material (Figure 5), whereas this does not occur as a rule for the pure PET. The PET samples containing 10 wt% (ε_b_ = 11 ± 6%) and 20 wt% (ε_b_ = 14 ± 4%) LDPE break quickly after neck initiation. The 80 wt% PET sample shows several marks of neck initiation. The ε_b_ of the 95 wt% PET blend is 33 ± 11%. Thus, compared to the PET/LLDPE blends, the PET/LDPE blends are less capable of plastic deformation. Large reductions in σ_y_ are again observed, compared to the plastically deformed virgin PET. The E values for these PET/LDPE blends deviate more from the additivity rule with increasing LDPE concentrations. 

##### LDPE/PS Blends

The PS-contaminated LDPE matrices show similar deformation to the pure LDPE material (Figure 5). However, ε_b_ decreases gradually, from 97 ± 4% for the homo material to 16 ± 6% for the 20 wt% PS-contaminated blend. The higher the content of PS contaminant, the more marks of potential neck initiation can be observed. E and σ_y_ increase significantly with increasing PS content.

The 50–95 wt% PS blends have ε_b_ values lower than 3%, and thus the samples show brittle breakage (Figure 5). As with the PS/LLDPE blends, crazing is not visible in the blend materials. σ_y_ shows a sharp decline as the LDPE content increases. Increasing concentrations of LDPE also cause a decrease in E, with the values for the 50–90 wt% blends being below the predictions made by the proportional rule.

#### 3.2.4. Mechanical Properties of HDPE Blends

##### HDPE/PA-6 Blends

The virgin HDPE polymer elongates to above 200% strain, whereby distinct necking occurs during the plastic deformation of the material. At 5 wt% (ε_b_ = 202 ± 69%) and 10 wt% (ε_b_ = 110 ± 49%) PA-6 contamination, this neck formation is still noticed, but fibrillation of the formed neck occurs (Figure 6). The HDPE/PA-6 blends with 20 wt% and 50 wt% PA-6 show brittle failure, with an ε_b_ value of 14 ± 1% and 7 ± 1%, respectively. The σ_y_ values for the 5–50 wt% contaminated PA-6 blends are close (within ±10%) to the value of the virgin HDPE. E values are slightly below the proportional line.

The blends with 80 wt% and 90 wt% PA-6 show a clear necking phenomenon, as observed during the deformation of the PA-6 homo material (Figure 6). However, ε_b_ is reduced from 234 ± 102% (pure PA-6) to 134 ± 36% for the 90 wt% PA-6 blend and 50 ± 8% for the 80 wt% PA-6 blend. The deformation of the blend with a PA-6 amount of 95 wt% is remarkable, as the blend material does not show distinct necking but instead deforms homogeneously over the length of the material. A higher ε_b_ (318 ± 71%) is measured for this particular blend. σ_y_ is declining sharply for increasing amounts of HDPE. E, on the other hand, follows additivity.

##### HDPE/PET Blends

The 5 wt% and 10 wt% PET-contaminated HDPE samples show necking, as with the pure HDPE, but, again, this neck is strongly fibrillated upon deformation (Figure 6). The specimens show a pronounced cup-cone fracture, achieving ε_b_ values of 364 ± 129% and 122 ± 39% for the 5 wt% and 10 wt% PET, respectively. For the 10 wt% PET blend material, the core of the material breaks quickly after neck initiation, while the side parts of the specimens show plastic deformation and macroscopic fibrillation. The blend containing 20 wt% PET shows similar deformation but fails much faster, with a more limited deformation of the side regions (ε_b_ = 10 ± 2%). The σ_y_ values of the 5–20 wt% PET blends remain at the level of the pure HDPE. E increases according to additivity.

The blends with 50 wt% and 80 wt% PET show a completely brittle failure (Figure 6). The material containing 80 wt% PET shows a fracture alongside a 45° plane. The 90 wt% PET material shows neck formation but fails quickly after neck initiation (ε_b_ = 14 ± 4%). Remarkable is the high elongation of the PET material containing 5 wt% HDPE with an ε_b_ of 364 ± 129%. This material initiates necking close to the dogbone grips and shows marks of this progressive neck. The PET matrices contaminated with HDPE weaken in terms of σ_y_ (compared to the yielded virgin PET samples) and E.

##### HDPE/PS Blends

Adding a PS contamination of 5 wt% and higher results in a brittle fracture of the tested HDPE matrix materials (Figure 6). The different blends show various marks where a neck could be initiated, but the materials break before an actual neck can be formed. The ε_b_ values drop for the 5 to 50 wt% PS blends, to 9 ± 2%, 7 ± 1%, 4 ± 1%, and 2 ± 0%, respectively. The σ_y_ values for these blends gradually decrease for increasing PS content. On the other hand, PS causes an increase in E.

When PS is the majority phase, a brittle failure is always observed. The measured ε_b_ values are even lower than those of the pure PS material. The values for σ_y_ decrease sharply with increasing amounts of HDPE. E follows the proportional rule.

#### 3.2.5. Mechanical Properties of PP Blends

##### PP/PA-6 Blends

In contrast to the pure PP material, no neck initiation is observed for the PP matrices contaminated with PA-6 (Figure 7). ε_b_ drops to a value between 10 and 20% for these blends. Stress whitening is noticed for these blends. σ_y_ decreases linearly from 36.9 ± 0.2 MPa for the pure PP, to 29.8 ± 0.4 MPa for the 20 wt% PA-6 blend. E follows additivity, and, for the 5 wt% PA-6 blend, a significantly higher value than this prediction is noted.

For the 90 wt% and 95 wt% PA-6 materials, a neck can be observed as in the pure PA-6 material (Figure 7). The 80 wt% PA-6 material shows an initiated neck, but the material fails before it can propagate. There is a negative impact on ε_b_ with increasing amounts of PP. Here, a decrease in σ_y_ is also observed. E, on the other hand, presents a synergistic effect for the 80–95 wt% PA-6 blends, attaining E values higher compared to the pure PA-6.

##### PP/PET Blends

As with the PA-6-contaminated PP matrices, brittle failure is observed for all tested combinations of the PP/PET blends (Figure 7). ε_b_ even drops below 10% for all these blends. σ_y_ decreases markedly for increasing PET content, but this is less steep than in the case of the PP/PA-6 blends. E values follow the additivity rule.

The 80–95 wt% PET blends show an initiated neck, but the material breaks quickly after this neck is formed (Figure 7). In comparison to pure PET samples, which show ductile deformation, a strong decrease in σ_y_ with increasing PO content is noticeable again. A synergistic effect for E can be observed for the 90 and 95 wt% PET blends, but this is less pronounced compared to the PA-6/PP combinations.

##### PP/PS Blends

The PP blend containing 5 wt% PS shows necking and attains a relatively high ε_b_ of 101 ± 30%. Upon addition of 10 wt% and 20 wt% PS, the ε_b_ drops to 17 ± 6% and 7 ± 1%, respectively. The 10 wt% PS specimens still show the initiation of necking, whereas the 20 wt% PS material already shows brittle breakage (Figure 7). σ_y_ values of the 5–50 wt% PS blends remain at the level of the pure PP material. E for these blends is within a 10% error of additivity.

The 80–95 wt% PS blends all show brittle fracture, yielding very low (<2%) ε_b_ values (Figure 7). The addition of PP to a PS matrix causes a decrease in σ_y_. The E values of these PS/PP blends decrease linearly according to the proportional rule. 

## 4. Discussion

### 4.1. Deformation of Contaminated LLDPE Matrix Blends

The LLDPE used is a polymer with mostly linear chains containing a significant number of short C4 side branches. As described in previous research, this polymer is able to deform to high strain values due to the underlying deformation mechanism of uniform shear yielding [23]. This mechanism, which operates in the crystalline lamellae of the polymer, prevails over the occurrence of plastic deformation of the amorphous fractions (e.g., via cavitation) for two reasons [43]. Firstly, the high proportion of these short side branches disrupts the regular chain folding process compared to a pure linear PE backbone [44], resulting in the formation of thinner lamellae during crystallization. Secondly, the presence of these regular side branches ensures the formation of a strong amorphous network by increasing the content of tie molecules (the molecules that form a physical connection between different crystalline lamellae) and the degree of entanglement in the amorphous phase [44,45,46]. 

**Figure 8:**
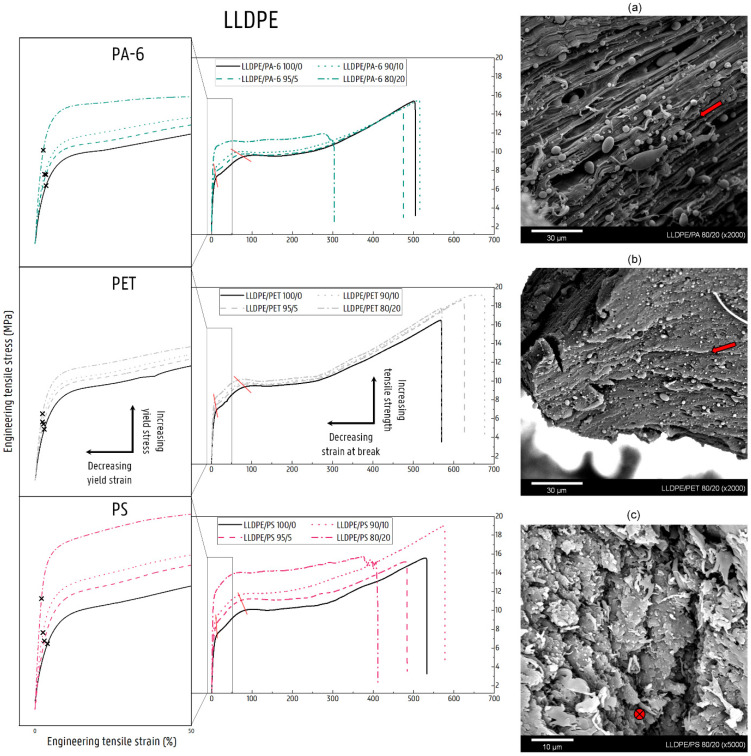
Stress–strain curves of the NPO-contaminated LLDPE matrices (0, 5, 10, and 20 wt% PA-6, PET, or PS). The 🗙 markings on the magnified panels indicate the 0.2% offset yield stress. The red lines indicate the double yield point phenomenon correlated with the occurrence of fine and coarse slip. The scanning electron microscopy (SEM) images show the area of rupture of the tensile-tested (deformed) binary blend specimens: (**a**) LLDPE/PA-6 80/20, (**b**) LLDPE/PET 80/20, (**c**) LLDPE/PS 80/20. The red arrow indicates the polymer flow.

The contamination of NPO has a clear reinforcing and stiffening effect on the blend material, as is also evident from the stress–strain curves (Figure 8). In the region of elastic deformation and thus at low strain values, the imposed loads are supported by both the LLDPE matrix and the more rigid NPO dispersion [47]. However, the linear trend of the proportional mixing rule is not observed due to the poor interfacial adhesion that exists between both polymer phases [1,48]. The lack of effective entanglements causes the stresses to be ineffectively transferred from the LLDPE matrix to the dispersed NPO, hence giving rise to only limited increases in E values. For the 20 wt% blends, a fourfold increase in E relative to the LLDPE is observed for the LLDPE/PA-6 and LLDPE/PS blends, compared to only a doubling for the PET-contaminated blend. The SEM images of the fracture surfaces of the tensile tested specimens in Figure 8a–c show the immiscible structures formed for the 80/20 wt% LLDPE/PA-6, LLDPE/PET, and LLDPE/PS blends, respectively. The LLDPE/PET 80/20 wt% blend shows good dispersion and distribution of the PET phase, while the other NPO-contaminated blends display a much coarser morphology. In these blends with PA-6 and PS contamination, partial continuity of the NPO phase is observed in addition to the larger NPO dispersions (Figure 8a,c). The coarser phase-separated microstructure, in comparison with the PET blend, is the result of the higher viscosity and lower melt elasticity of PS and PA-6 versus PET and the dynamic mixing conditions during injection molding, which favor the coalescence of the PS or PA-6 phases [17,49,50,51,52,53,54,55,56]. Rotational rheology data on complex viscosity, storage modulus, and loss modulus are given in the Supplementary Information (Figure S5). The continuity provides an interpenetrating effect of the NPO phase that explains the higher stiffness of the LLDPE/PA-6 and LLDPE/PS 80/20 wt% blends [57,58].

The weak interfacial adhesion that exists between the LLDPE polymer and the NPO phase also exerts its influence on the plastic deformation of the blend material. Thus, at higher elongation, in addition to the competition between the shearing of crystalline lamellae and the formation of cavities in the amorphous phase of the matrix, a third deformation mechanism will come into play in the blend materials, namely the decohesion of the phase-separated microstructure. The SEM micrographs in Figure 8a–c indicate that in the NPO-contaminated LLDPE blends, the plastic deformation will be an interplay of the decohesion of the NPO phase and the shear yielding of the LLDPE matrix. The NPO droplets present act as stress concentrators and will be the trigger for increased local stresses [59]. Under the influence of this higher triaxial stress state, the polymer phases decohere due to the weak interfacial adhesion existing between the LLDPE and NPO polymers [1,48]. The created decohesion microvoids alter the local stress state, which will subsequently induce the onset of the plastic deformation of the LLDPE crystalline lamellae [32]. The recorded stress values never exceed the σ_y_ values of any of the NPO materials, meaning that the NPO dispersions are not subject to plastic deformation, which is confirmed by the SEM micrographs shown (Figure 8). Figure 8a,b clearly show the elongated LLDPE matrix, whereas the NPO dispersions do not show plastic deformation and thus remain spherical of shape. Hence, following decohesion, the LLDPE matrix will start to deform independently of the dispersed NPO phase owing to its strong polymer network of tie molecules and entanglements in the amorphous regions. The shear yielding thus propagates in the ductile LLDPE matrix, while preventing further opening of the decohesion voids under the influence of the external stress. Despite the LLDPE-containing NPO contamination, the blend materials still show homogeneous deformation (without necking) to high strain values (>300%) at contamination levels up to (and including) 20 wt%.

Shear yielding is a sequence of fine and coarse crystallographic slip processes, as evidenced by the occurrence of a double yield point in the stress–strain curves of the LLDPE material [60,61,62,63]. These points are not to be confused with the zero-slope or 0.2% offset yield stress. The two points marked by the red lines in Figure 8 indicate on the stress–strain curves the onset of fine slip and coarse slip, respectively [62]. For most of the LLDPE matrix blends, except the LLDPE blends with 20 wt% PA-6 and PS, this double yield point is still clearly observed in the stress–strain curves of the blend materials (Figure 8). For these respective blends, both fine and coarse slip are encountered at smaller strain values compared to the virgin LLDPE, due to the higher local stress state induced by the presence of the NPO dispersion. In the case of these blends, after disintegration of the crystalline blocks, disentanglement and alignment of the amorphous LLDPE chains will occur, giving rise to the strain hardening behavior, as also found in the pure LLDPE. The increased stress state in the blend can even lead to increased values of ε_b_ because the LLDPE chains are able to slip past each other to a higher extent under the elevated stress level. In contrast, the addition of 20 wt% PA-6 or PS causes a more diffuse transition from fine to coarse slip and significantly disrupts the strain hardening behavior, resulting in a clear drop in ε_b_ (Figure 8). The coalescence of the dispersed PA-6 or PS leads to larger phase domains and consequently to larger voids upon decohesion (Figure 8a,c). In addition, a remarkable increase in stress level is noticeable for these blends. The excessive stress level will cause the voids to open up, leading to cracks, which in turn will accelerate material failure. Hence, the importance of a fine dispersion, as seen for the LLDPE/PET 80/20 wt% blend in Figure 8b, becomes clear. It allows the LLDPE matrix to better dissipate the triaxial stress induced by the NPO contamination and to continue to undergo the homogeneous deformation typical for the pure LLDPE material, even at 20 wt% NPO contamination. At higher concentrations of NPO, further continuity of this polymer will be expected. For the blends with equal amounts of NPO and LLDPE, the switchover to an NPO matrix occurs (Figure 4), and the LLDPE as a minority polymer will thus affect the deformation of the respective NPO matrix.

### 4.2. Deformation of Contaminated LDPE Matrix Blends

In contrast to the short, regular, and identical chain branches of LLDPE, the structure of LDPE is defined by the presence of irregular long-chain branching, which may in turn contain short side branches. As with LLDPE, the side branches will disturb the formation of the crystalline structures, resulting in a low-crystalline material with imperfect crystalline lamellae. The long-chain branches are assumed to reach lengths greater than the critical entanglement molecular weight (for polyethylene) [39,64] and are prone to the formation of entanglements, already in the melt phase. These entanglements formed in the melt phase are preserved upon crystallization, as disentanglement is prevented due to the reduced chain diffusion of the long side branches containing the LDPE backbone [44]. Interlocking of the entanglements and tie molecules is further enhanced by the rapid quenching in the cold mold [44,65,66,67]. Long side chains increase the relaxation time of the individual LDPE chains, resulting in less time for the rearrangement of the individual LDPE molecules [44,68]. This slow rearrangement of the LDPE chains favors nucleation during crystallization and has a positive influence on the amount of tie molecules [44]. However, the smaller radius of gyration (less coil overlapping in the melt) of a branched polymer will have a negative impact on the number of intercrystalline links compared to more linear chains [44,69]. As a result, the crystalline structure of LDPE is characterized by more intrachain entanglements and fewer interchain entanglements. Relative to LLDPE, the crystalline blocks of LDPE are therefore less interconnected. This is reflected in the stress–strain curve of the mono LDPE, where the material behaves in a less ductile manner than the LLDPE polymer used (Figure 3). The LDPE will plastically deform via shear yielding with the initiation of a local neck, which then fails to propagate before material failure [23]. The entanglements in the LDPE polymer network and the long side branches limit the ability of the LDPE chains to move past each other, resulting in a higher stress plateau as opposed to the LLDPE used.

**Figure 9:**
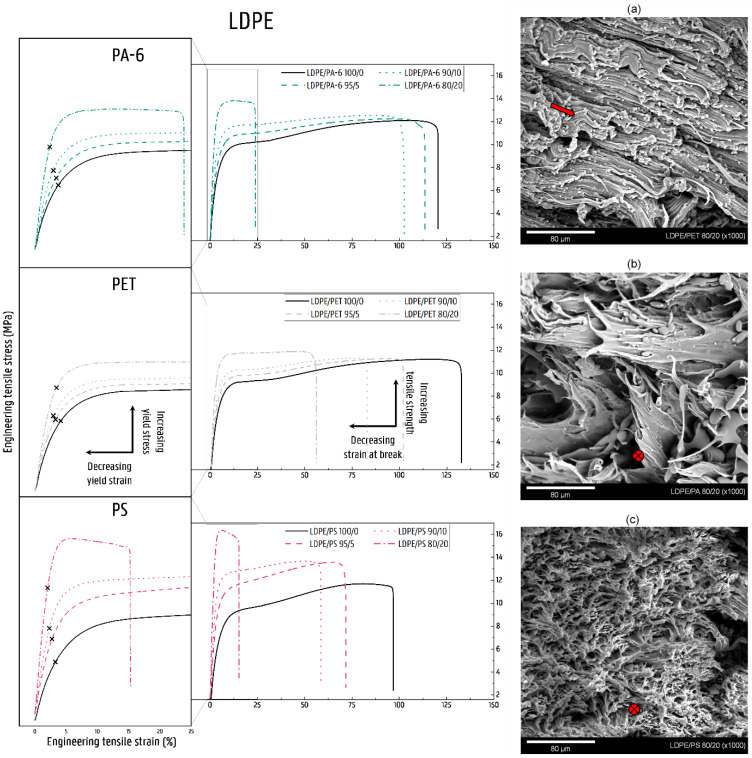
Stress–strain curves of the NPO-contaminated LDPE matrices (0, 5, 10, and 20 wt% PA-6, PET, or PS). The scanning electron microscopy (SEM) images show the area of rupture of the tensile-tested (deformed) binary blend specimens: (**a**) LDPE/PET 80/20, (**b**) LDPE/PA 80/20, (**c**) LDPE/PS 80/20. The red arrow indicates the polymer flow.

The stress–strain diagrams of the LDPE matrices (Figure 9) once again confirm the increase in stiffness and strength after the addition of an NPO contaminant. At low strains, in the elastic deformation region, the stresses are poorly transferred from the LDPE matrix to the more rigid NPO dispersion, as is the case with LLDPE. Here, the overall higher increase in E values for the PS-contaminated blends indicates a better interaction between LDPE and the dispersed PS compared to the polar polymers PA-6 and PET. In addition, increases are measured in the crystallinity of the NPO-contaminated LDPE matrices (Figure 1), which generally have a positive influence on E and σ_y_ [47]. Especially for the addition of a PS phase, a significant increase in the crystallinity of the LDPE matrix is measured (see Figure 1). The higher degree of crystallinity of the LDPE matrix contributes to the increasing E and σ_y_, whose values approximate more closely the additivity rule for the LDPE/PS blends. Eventually, the phase-separated microstructure will also be reflected in the increase in the values of the functional properties E and σ_y_ of the blend materials. At a contamination level of 20 wt%, the NPO polymers will form larger domains and even partly co-continuous structures in the case of the PA-6- and PS-contaminated blends (Figure 9b,c). The SEM images in Figure 9b,c depict how the LDPE matrix strands are forced to deform around the NPO phase, indicating the interpenetrating nature of PA-6 and PS in these polymer blends. The large NPO domains thus result in higher E and σ_y_. The dynamic conditions during injection molding and the higher melt elasticity of PA-6 and certainly PS compared to PET cause this morphology to occur (Figure S5 in Supplementary Information). The 80/20 wt% LDPE/PET blend shows a fine dispersion, as can be seen on the SEM micrograph given in Figure 9a.

At higher strains, the elongation of the blend materials is determined by the plastic deformation of the LDPE matrix, since the stress level is insufficient to allow the NPO phase to deform plastically. The presence of the NPO phase causes locally concentrated stresses in the LDPE matrix. Under the influence of this elevated triaxial stress state, decohesion between the NPO phase and the deforming LDPE material will occur upon further stretching. The resulting microvoids at the interphase in turn initiate locally the crystallographic slip processes of the LDPE lamellae. In addition to the plastic deformation caused by crystallographic slip processes, the network of amorphous LDPE chains exerts resistance to the ongoing deformation. Upon stretching, the entanglements of these amorphous chains and their long side branches cause the mild upward trend seen in the stress signal of the tensile curves starting at strain levels around 25% (Figure 9). It is also visible from these diagrams that the higher stress levels induced by the presence of the rigid NPO phase initiate plastic deformation at lower strains. The better-distributed and finer PET results in the maximum measured stress being lower compared to the coarser dispersions of PA-6 and PS.

Relatively high ε_b_ values (>50%) are still obtained for blends containing 5 and 10 wt% NPO, where the LDPE matrix is able to deform pseudo-uniformly around the voids created by decohesion. This deformation behavior is favored by the good dispersion and distribution of the contamination polymer in the LDPE matrix. However, in contrast to the LLDPE material, there is a significant decrease in ε_b_ noticed for all the NPO-contaminated LDPE blends compared to the neat LDPE. ε_b_ drops more dramatically as the concentration of NPO increases. As mentioned, the crystalline LDPE lamellae are less interconnected by intercrystalline links. Consequently, the LDPE material is more susceptible to tearing open around the NPO phase present. Under the elevated and increasing stress level, the voids will continue to grow in the direction of the load. The elongated opened voids can be recognized in Figure 9a. The deformed LDPE is hereby divided into strands under the influence of the progressive tearing and coalescence of the voids. The crystalline lamellae will further disintegrate under the influence of the increasing stress, followed by alignment and slip of the LDPE amorphous chains. This disentanglement of the polymer chains is, however, hampered by the long side branches of the LDPE. At this neck initiation, the LDPE will no longer be able to bear the imposed load, in which the failure of the blend material is intensified by the higher stress levels and the reduced cross-sectional area due to the presence of the opened-up voids. In the case of the PA-6-contaminated blends, the material succeeds in propagating its shear yielding in the LDPE-rich zones throughout the specimen. For the LDPE/PET and LDPE/PS blends with 5 and 10 wt% NPO contaminant, the drops in ε_b_ are more drastic. The good distribution of the PET phase facilitates the convergence of the ruptured voids and thus leads to faster failure of the blend material. In the case of the 95/5 wt% and 90/10 wt% LDPE/PS blends, the coarser PS dispersion will weaken the blend material, where it causes larger decohesion voids to occur and thus results in reduced ε_b_ values. The strong decreases in ε_b_ for the 80/20 wt% LDPE/PA-6 and LDPE/PS blends are due to the occurrence of a co-continuous structure. As can be seen in Figure 9b,c, the deformation of the LDPE matrix is hindered by the interpenetrating NPO phase. In addition, material failure of the LDPE ligaments deforming around the NPO phase occurs more rapidly due to the overall stress levels being elevated by the rigid NPO polymer.

### 4.3. Deformation of Contaminated HDPE Matrix Blends

The linear structure with the absence of side branches allows the HDPE chains to pack into denser and more perfect crystalline lamellae [39]. HDPE thus possesses a higher degree of crystallinity, making it stiffer and stronger compared to the low-crystalline polyethylenes LDPE and LLDPE. The thicker crystalline lamellae make the HDPE material more sensitive to the occurrence of cavitation in the amorphous regions of the polymer [70,71]. Generally, the deformation of HDPE progresses via a shear yielding process [24,34,35] in which the clear propagation of a neck occurs [72,73,74]. 

**Figure 10:**
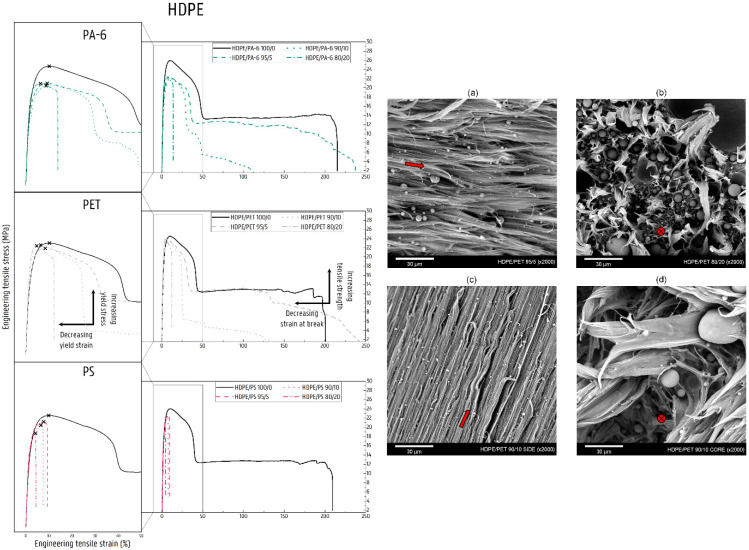
Stress–strain curves of the NPO-contaminated HDPE matrices (0, 5, 10, and 20 wt% PA-6, PET, or PS). The scanning electron microscopy (SEM) images show the area of rupture of the tensile-tested (deformed) binary blend specimens: (**a**) HDPE/PET 95/5, (**b**) HDPE/PET 80/20, (**c**) HDPE/PET 90/10 side region, and (**d**) HDPE/PET 90/10 core region. The red arrow indicates the polymer flow.

The stiffness of the NPO-contaminated HDPE matrices shows an upward trend with increasing NPO content. Compared to the low-crystalline PEs, however, the increase is more limited because the HDPE polymer has a smaller and more restricted amorphous fraction, which accounts for the elastic deformation. Thus, the initial E value of HDPE is higher and the stiffer NPO phase will, additional to the effect of incompatibility, only partly contribute to a stiffness increase in the blend material. The immiscibility between the HDPE matrix and the dispersed NPO polymer causes the stress transfer between the two polymer phases to be low. Subsequent to elastic deformation of the blend, the HDPE matrix will decohere from the NPO phase, resulting in the formation of microvoids at the weak interphase. This relieves the triaxial stresses induced by the NPO dispersion, marking the onset of the plastic deformation of the HDPE matrix. As with LDPE- and LLDPE-contaminated matrices, the local stress concentration due to the presence of an NPO polymer triggers local plastic deformation in the HDPE matrix at lower strain values. The local initiation of the plastic deformation extends to the surrounding HDPE material, resulting in a reduction in the σ_y_ values of each of the contaminated HDPE matrix blends compared to the neat HDPE (Figure 10). The values of the blends containing PET remain close to the level of the pure material. Despite the similar trends observed via DSC analysis with the HDPE/PA-6 blends, it is assumed that the orientation and/or the degree of crystal perfection is higher in these HDPE/PET blends. The SEM images (Figure 10) show the denser packing of the PET droplets in the core of the test sample, which are more restrictive to the plastic deformation of the HDPE matrix. This results in a higher stiffening and strengthening effect in these HDPE/PET blends. For the PS-contaminated blends, the morphology is coarser and less well-distributed (see SEM images in Figure S6 in Supplementary Information), and, consequently, the HDPE-PS blend materials fail before reaching a yield plateau, resulting in lower σ_y_ values. 

The plastic deformation of the HDPE matrix is thus a combination of the slip processes in the HDPE lamellae, on the one hand, and the progressive longitudinal opening of the microvoids created by decohesion at the phase separation on the other hand. Higher stresses compared to the low-crystalline PEs are established in the stiffer HDPE matrix, which reinforces the growth of the formed voids in the direction of the load. Moreover, the HDPE is insufficiently tough and thus unable to resist this progressive tearing. Upon further stretching, the deforming HDPE matrix will be inclined to initiate the formation of a neck by means of coarse slip in the crystalline lamellae. In the case of the HDPE with 5 wt% and 10 wt% PA-6 and PET, the material is also able to propagate its deformation into a stable neck. In these blends, upon propagation of this necking, the crystalline blocks undergo further rearrangement and fragmentation into fibril structures oriented in the load direction [72,73,74]. This is accompanied by the extensive macroscopic fibrillation caused by the continued rupture of the decohesion voids (Figure 10a,c). Eventually, the separated HDPE strands are no longer able to withstand the imposed force, and, consequently, the material fails. For the 10 wt% HDPE/PET and the HDPE/PA-6 blends, quick fracture of the core section of the specimen is observed, while the side regions show high elongation. The injection molding processing results in a difference in phase morphology between the skin and core sections, in which a fine dispersion of the PET phase develops in the skin of the test specimen [75,76,77], as is also evident in Figure 10c. In addition, the material is subject to higher shear and cooling along the mold wall. This leads to a higher orientation and reduced crystallinity of the matrix material, allowing the shear yielding process to take place in the side regions of the samples. On the other hand, the more crystalline core of the injection-molded sample is more prone to cavitation [70,71]. However, at higher strain levels, debonding of the HDPE and PET phases will occur again. Both processes ultimately lead to the growth and connection of the created voids and cause the core section to break rapidly (Figure 10d). With the core broken, the imposed load must be borne by the plastically deforming side regions, which will fibrillate faster and result in lower ε_b_ values for these blend materials. Onset of this disparity is also observed for the addition of 20 wt% PET in HDPE, but the PET phase is most likely too abundant (Figure 10b), leading to a low ε_b_. Likewise, in the case of the 80/20 wt% HDPE/PA-6 blend, the dispersed NPO phase domains are too numerous, causing the voids formed during decohesion to coalesce (Figure S6 in Supplementary Information). Further crack opening and merging leads to failure without neck initiation. This is also the case for each of the HDPE/PS blends investigated. Here, a drastic drop in ε_b_ is already observed for the lower percentages of added PS. This can be attributed to the lesser dispersion and distribution of the more viscous PS material (Figure S6 in Supplementary Information).

### 4.4. Deformation of Contaminated PP Matrix Blends

Pure PP, similar to HDPE, is a PO with a strongly crystalline structure. The uniform isotactic architecture of the investigated PP allows packing of the polymer chains into strong chain-folded crystalline structures, which are mostly spherulitic [78]. The presence of the methyl group results in a stiffer and stronger material compared to PE. Pure PP undergoes a similar deformation mechanism under tensile load as the HDPE material, namely shear yielding with the initiation and propagation of a distinct neck. 

**Figure 11:**
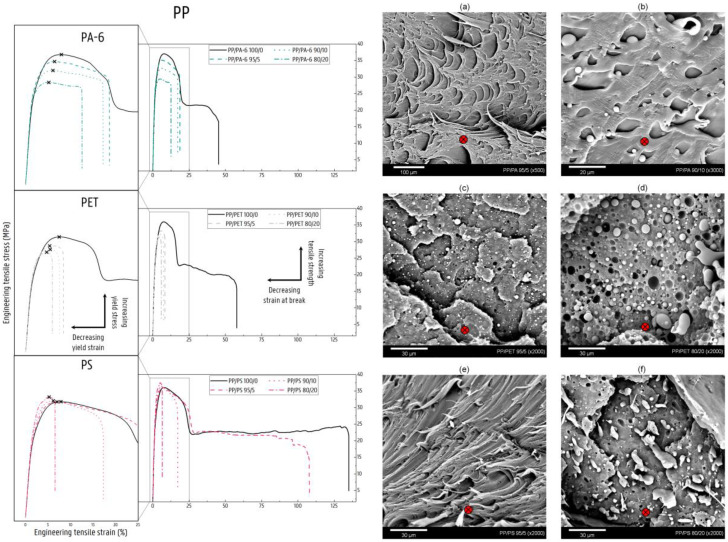
Stress–strain curves of the NPO-contaminated PP matrices (0, 5, 10, and 20 wt% PA-6, PET, or PS). The scanning electron microscopy (SEM) images show the area of rupture of the tensile-tested (deformed) binary blend specimens: (**a**) PP/PA 95/5, (**b**) PP/PA 90/10, (**c**) PP/PET 95/5, (**d**) PP/PET 80/20, (**e**) PP/PS 95/5, and (**f**) PP/PS 80/20. The red arrow indicates the polymer flow.

As with the HDPE material, the high crystalline structure and, additionally, the more rigid chain structure of the PP will largely account for the stiffness of the blend materials. The slight increase in the E value for each of the investigated blends suggests, however, a contribution of the stiffer NPO in the low-strain region. Poor interfacial adhesion hampers the stress transfer from PP matrix to NPO phase. In the case of the PP/PS blends, there is a clear increase in the crystallinity of the PP matrix (Figure 1). The E values under the proportional rule, however, indicate the greater but less perfect formation of PP crystals. As also noticed for the other PO matrices, the presence of the NPO phase initiates the plastic deformation of the PP matrix at lower ε_y_ values. In the case of the PP/PA-6 and the PP/PET blends, a gradual decrease in σ_y_ with increasing NPO content can also be observed from the tensile curves given in Figure 11. The introduction of locally elevated stresses at the weak interphase due to the low ε_y_ values of the pure NPO polymers will cause the PP matrix to detach from the dispersed NPO phase upon further elongation. The PP matrix will therefore dominate the plastic deformation of the different PP/NPO blends. The formation of microcavities during the decohesion marks the initiation of plastic deformation via shear yielding of the PP material at lower σ_y_ and ε_y_ values compared with the neat PP. With higher amounts of NPO, the size of this NPO phase also increases, which in turn enlarges the region of elevated stress in the surrounding PP material, and thus can lower the critical stress required for the initiation of shear yielding of the PP matrix [59,79,80]. Low levels of NPO will typically entail a wider distance between individual NPO domains, so the PP ligaments between the NPO phases will be less likely to engage in plastic deformation [59,81]. In addition, the presence of the NPO phase reduces the cross-sectional area that is responsible for bearing the imposed force, which may also contribute to the decline in strength for increasing amounts of NPO. 

Nonetheless, the plastic deformation via slip processes of the PP crystalline lamellae is limited for the PA-6- and PET-contaminated PP matrices. The relatively stiff PP is not able to compensate for the expansion of the decohesion voids with toughening mechanisms. The cavities grow perpendicular to the load direction and develop into cracks, which propagate from the NPO droplet to adjacent droplets, eventually leading to material fracture. Here, the PP material is not able to propagate in a stable neck. Regarding the PP/PET and PP/PA-6 blends, consistently lower ε_b_ values are observed for the blends with PET contamination compared to the PP/PA-6 blends (Figure 11). The explanation for this lies in the fact that the PET dispersion is better distributed throughout the specimens, resulting in accelerated formation of the brittle fracture surface between the more closely packed PET dispersions (Figure 11c,d). In the case of the PA-6-contaminated PP matrices, more plastic deformation can be observed due to the propagation of shear yielding in the more PP-rich zones of the specimens. This is visible on the SEM images given in Figure 11a,b (PP-PA-6 90/10 ×5000). Figure 11b shows the limited plastic deformation of the PP matrix with the growth of the voids perpendicular to the tensile direction.

In the blends containing 5 wt% and 10 wt% PS, the propagation of necking is still observed. It is assumed that the blend material can initiate a neck on a PP-rich zone in the specimen. This is due to the fact that the PS phase is poorly distributed throughout the PP matrix compared to the PET and PA-6 polymers. Additionally, the crystallinity of the PP matrix is higher in these PS-contaminated blends compared to the neat PP. However, the DSC signals for these blend materials reveal that the PP melting peaks shift to lower temperatures (see DSC signals in Supplementary Information (Figure S4)). Therefore, it is assumed that the crystals are smaller but more numerous. These weaker crystals are more susceptible to the onset of plastic deformation. In the 95/5 wt% PP/PS blend, the PP crystalline regions will thus deform plastically, as evidenced by the ductile fracture surface in Figure 11e. The sample containing 10 wt% PS also shows the transition to a stable neck; however, crack propagation will proceed following interfacial decohesion. Since shear yielding occurs in the PP-rich zones without a major influence from the PS phase present, the σ_y_ values for these blends are retained at the level of the pure PP material (Figure 11). The higher σ_y_ in the 80/20 wt% PP/PS blend is again a consequence of the contribution of the PS polymer by the partial continuity of this PS phase through the blend material (Figure 11f). Under crack propagation, the PP material will break, and the brittle minority PS phase is not able to support the applied force, consequently resulting in material failure.

**Figure 12:**
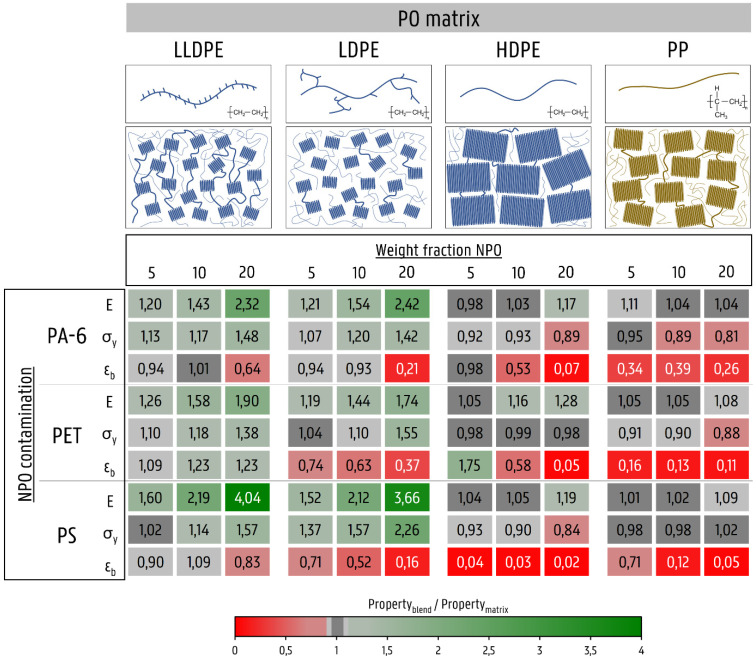
Overview of elastic modulus, yield strength, and strain at break for the PO matrices contaminated with PA-6, PET, and PS. The values shown are the ratios of the property of the blend to the property of the neat PO matrix.

The abovementioned results and discussion are summarized in Figure 12. In the Supplementary Information, Table S1 is included, which lists the absolute values of the mechanical properties of all mono and blend materials. Figure 12 clearly shows the difference in the change in properties between the low-crystalline polymers, LLDPE and LDPE, and the high-crystalline polymers, HDPE and PP. The flexible LLDPE and LDPE matrices show a strong increase in stiffness and strength, especially when 20 wt% NPO is added. For the stiffer HDPE and PP matrices, the increases in E are milder, and decreases in zero slope σ_y_ are observed. 

The PO matrix structure of the crystalline phase and the interconnections with the amorphous fractions largely determine whether the blend material is able to deform plastically to high elongation, despite the presence of the NPO contaminant. The high ductility of the contaminated LLDPE matrices is due to the many interlamellar links and the linear structure of the polymer. Likewise, LDPE manages to deform plastically, similar to the neat polymer. However, the lack of intercrystalline links will cause ε_b_ to drop with increasing NPO content. The high-crystalline HDPE and PP polymers fail to prevent the tearing of the decohesion voids, causing fibrillation or even brittle fracture.

The relative variations among the used NPO materials, PA-6, PET, and PS, are mainly due to the degree of dispersion and distribution throughout the PO matrix. This, of course, strongly depends on the rheological properties and the difference thereof for the matrix and minority phase. At higher concentrations of NPO polymer, the size of the dispersed phase increases, as well as the probability of partial continuity, which can have a drastic impact on the ductility of the PO matrix.

## 5. Conclusions

This work investigated the changes in mechanical properties and occurring deformation mechanisms in binary PO/NPO blends compared to the neat PO materials under tensile loading. For this purpose, blend series spanning the entire composition range were produced via injection molding by blending four POs (LLDPE, LDPE, HDPE, and PP) with three different NPOs (PA-6, PET, and PS).

SEM imaging showed that each of the investigated blends exhibited a distinct phase-separated morphology in which the degree of dispersion and distribution of the NPO phase was linked to the rheology and processing characteristics of the matrix and minority phases. The immiscibility between PO and NPO was reflected in the fact that the properties did not simply follow a proportionality rule.

Each of the four POs used exhibited a baseline deformation behavior, whereby plastic deformation of the crystalline lamellae occurred via shear yielding. The impact of NPO contamination on the deformation behavior of the PO matrix was strongly linked to the degree of dispersion and distribution of the minority phase and the potential for interconnections between the crystalline and amorphous regions of the PO to propagate its plastic deformation.

Clear differences were observed between the low-crystalline LLDPE and LDPE, on the one hand, and the high-crystalline HDPE and PP on the other. At very low strains, in the elastic deformation region, a dispersed NPO phase caused a gradual increase in the elastic modulus of the PO matrix with increasing amounts of NPO. Due to the relatively high stiffness and high degree of crystallinity of HDPE and PP, these increases were limited compared to LLDPE and LDPE. The stiffer and stronger NPO materials increased the strength of the low-crystalline LLDPE and LDPE. In contrast, a reduction in strength was found in the HDPE and PP materials.

The property most affected by the presence of NPO contamination was the ductility or total plastic deformation that the material could undergo prior to failure. Despite high NPO levels (20 wt%), the LLDPE material exhibited ductile deformation behavior, with ε_b_ values above 300%. The crystalline domains in LLDPE were strongly interconnected by the amorphous polymer network. Moreover, due to the linear structure with regular and short branches, the LLDPE chains were able to slide past each other, reaching high elongations. LDPE also showed ductile behavior. However, LDPE has less intercrystalline links due to the branched structure of the polymer. Under the influence of increasing NPO amounts, gradual decreases in ε_b_ were observed. The high-crystalline matrices HDPE and PP experienced the most drastic impact on their ductility. At low amounts of NPO (5 wt%), ductile deformation behavior was still found for the contaminated HDPE matrix. However, higher NPO levels, and poor distribution thereof, led to brittle failure. In the stiffer PP material, embrittlement already occurred at contaminant levels of as little as 5 wt% NPO.

Research into the altered deformation behavior is essential with a view to the mechanical recycling of these cross-contaminated plastic waste streams. Real plastic waste streams, however, generally contain multiple plastic impurities, as well as non-polymeric contaminants (metals, wood, paper). It remains to be seen how these complex (ternary, quaternary, etc.) blends behave and which polymeric contaminants behave either synergistically or antagonistically. Future research should therefore focus on the influence of various contaminants on the properties of a recycled plastic material. In this way, conclusions based on virgin polymer blends can be extended to predicting the properties of real waste plastic feedstocks. The resulting mechanical properties will be of great importance in the proper assessment of the technical quality for application in new products.

## Figures and Tables

**Table 1:** Used polymers; data obtained from the respective technical datasheets.

	Material	Grade	Producer	MFI (g/10 min) *	Typical Use
PO	LLDPE	Exceed^TM^ 1012HA	ExxonMobil	1.0	Film
	LDPE	LD150AC	ExxonMobil	0.8	Film
	HDPE	25055E	Dow Chemical Company	25.0	Housewares, food containers, toys
	PP	PP6272NE1	ExxonMobil	2.8	Cups, containers
NPO	PET	Lighter C93	Equipolymers	-	Beverage and food containers
	PA-6	Akulon F230C	DSM	-	Film, injection-molded articles
	PS	Styrolution PS 158K	INEOS	3.0	Household goods, containers, expanded sheet and film

* Melt flow index (MFI)—PE: 190 °C, 2.16 kg; PP: 230 °C, 2.16 kg; PS: 200 °C, 5.0 kg.

**Table 2:** Used temperature profiles in injection molding.

Blend Combination	Temperature Profile (°C)
PET + PO	250–260–270–280
PA-6 + PO	245–255–265–275
PS + PP	200–210–220–230
PS + LLDPE/LDPE/HDPE	170–180–190–200

**Table 3:** Crystallinity and mechanical properties of the materials used, given for each processing temperature.

	Material	T_processing_ (°C)	Χ_c_ (%)	E (MPa)	σ_y_ (MPa)	ε_y_ (%)	ε_b_ (%)
NPO	PET	280	27.1	2337 ± 218	49.4 ± 15.7	3.07 ± 1.59	2.9 ± 2.5 *
	PA-6	275	28.5	2260 ± 60	62.1 ± 0.4	4.09 ± 0.03	234 ± 102
	PS	200	0	3084 ± 42	52.5 ± 0.6	4.33 ± 0.17	5.2 ± 0.6
		230	0	3202 ± 37	49.7 ± 0.3	3.62 ± 0.07	4.6 ± 0.4
PO	LLDPE	200	28.9	97 ± 3	5.3 ± 0.1	4.00 ± 0.19	533 ± 9
		275	28.0	109 ± 8	5.2 ± 0.2	3.51 ± 0.35	507 ± 18
		280	29.0	120 ± 4	4.4 ± 0.1	2.96 ± 0.19	569 ± 7
	LDPE	200	34.2	165 ± 9	5.5 ± 0.3	3.20 ± 0.28	97 ± 4
		275	33.5	154 ± 5	7.1 ± 0.1	3.73 ± 0.12	120 ± 2
		280	31.9	173 ± 37	6.5 ± 0.6	4.01 ± 0.88	132 ± 5
	HDPE	200	64.4	984 ± 14	24.2 ± 0.3	10.29 ± 0.71	220 ± 13
		275	64.1	893 ± 61	25.0 ± 0.5	10.30 ± 0.21	206 ± 32
		280	65.6	868 ± 19	24.4 ± 0.2	10.29 ± 0.08	208 ± 33
	PP	230	46.5	1706 ± 29	36.8 ± 0.3	7.87 ± 0.07	142 ± 20
		275	45.2	1672 ± 30	36.9 ± 0.2	7.90 ± 0.10	45 ± 9
		280	47.8	1745 ± 32	36.0 ± 0.1	7.59 ± 0.07	53 ± 10

* Average ε_b_ of the PET samples that showed brittle break.

## Supplementary Materials

The following supporting information can be downloaded at: https://www.mdpi.com/article/10.3390/polym14020239/s1, Figure S1: DSC signals of the NPO-contaminated LLDPE matrices; Figure S2: DSC signals of the NPO-contaminated LDPE matrices; Figure S3: DSC signals of the NPO-contaminated HDPE matrices; Figure S4: DSC signals of the NPO-contaminated PP matrices; Figure S5: Rotational rheology results for the mono materials used: (a) complex viscosity, (b) storage modulus, and (c) loss modulus; Figure S6: Scanning electron microscopy (SEM) images of the area of rupture from the tensile-tested (deformed) binary blend specimens: (a) HDPE/PA 95/5, (b) HDPE/PA 90/10, (c) HDPE/PA 80/20, (d) HDPE/PS 95/5, (e) HDPE/PS 90/10, and (f) HDPE/PS 80/20. The red arrow indicates the polymer flow.

## Abbreviations and Symbols

**Abbreviations**	
DSC	differential scanning calorimetry
HDPE	high-density polyethylene
LD	longitudinal direction
LDPE	low-density polyethylene
LLDPE	linear low-density polyethylene
MFI	melt flow index
NPO	non-polyolefin
PA-6	polyamide-6
PET	polyethylene terephthalate
PO	polyolefine
PP	polypropylene
PS	polystyrene
SEM	scanning electron microscopy
TD	transversal direction
**Symbols**	
°C	degrees Celsius
E	elastic modulus
J/g	joule per gram
kN	kilonewton
kV	kilovolt
mm	millimeter
mm/min	millimeter per minute
MPa	megapascal
N	newton
T_processing_	processing temperature
wt%	weight fraction
$\Delta H_{m}^{0}$	melting enthalpy of the 100% crystalline polymer
Δ*H_cc_*	cold crystallization enthalpy
Δ*H_m_*	melting enthalpy
ε_b_	strain at break
ε_y_	strain at yield
σ_y_	yield strength
Χ_c_	degree of crystallinity
