# Supplementary material for: Macromolecular Insights into the Altered Mechanical Deformation Mechanisms of Non-Polyolefin Contaminated Polyolefins
Source: Polymers (Basel). 2022 Jan 7;14(2):239. doi: 10.3390/polym14020239 (PMC8779932; doi:10.3390/polym14020239)
Supplement: Supplementary file 1 [file polymers-14-00239-s001.zip › polymers-1511383-supplementary.pdf]

SUPPLEMENTARY INFORMATION

Macromolecular insights into the altered mechanical deformation mechanisms of non-polyolefin contaminated polyolefins

***Ruben Demets*[1,2,3]*, Marie Grodent*[4]*, Karen Van Kets*[2,3]*, Steven De Meester*[2] *and Kim Ragaert*[1]**

[1] Circular Plastics, Department of Circular Chemical Engineering, Faculty of Sciences and Engineering, Maastricht University, Urmonderbaan 22, 6162 Geleen, The Netherlands

[2] Laboratory for Circular Process Engineering (LCPE), Department of Green Chemistry and Technology, Faculty of Bioscience Engineering, Ghent University – Campus Kortrijk, Sint-Martens-Latemlaan 2B/5, 8500 Kortrijk, Belgium

[3] DPI, P.O. Box 902, 5600 AX Eindhoven, the Netherlands

[4] Centre for Polymer and Material Technologies (CPMT), Department of Materials, Textiles and Chemical Engineering, Faculty of Engineering and Architecture, Ghent University, Technologiepark 130, B-9052 Zwijnaarde, Belgium

1. DSC diagrams of the NPO contaminated PO matrices

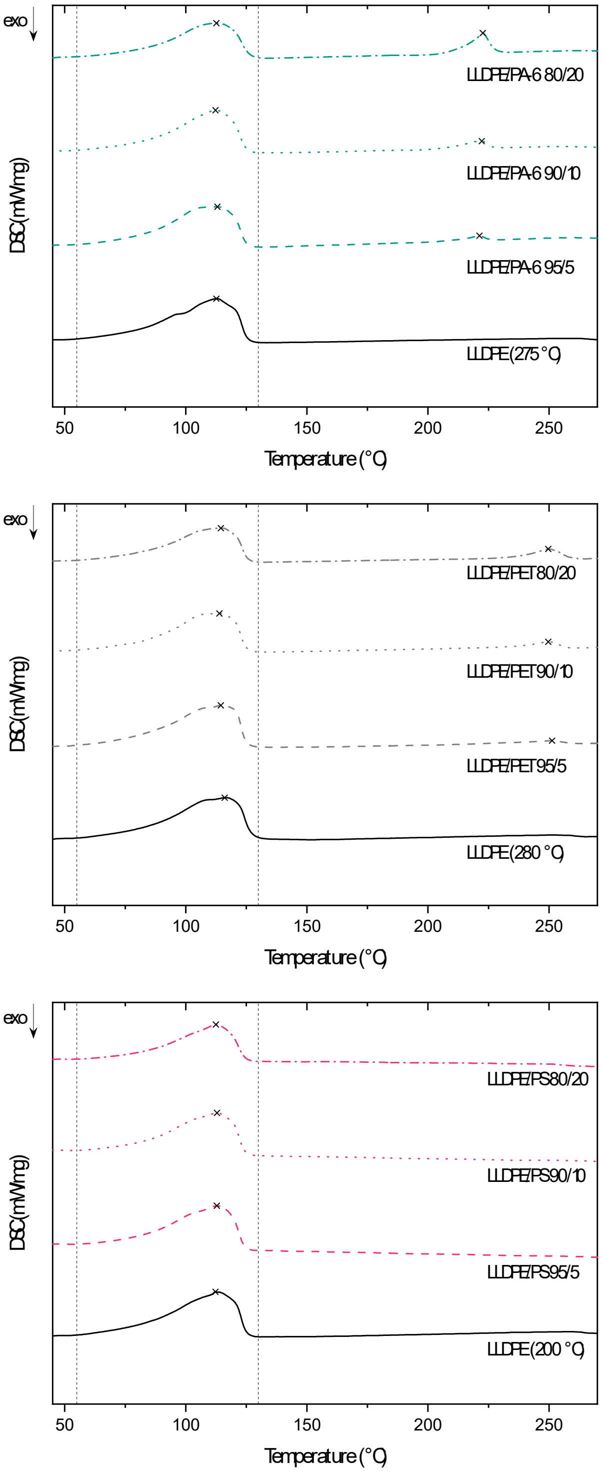

Figure S1: DSC signals of the NPO contaminated LLDPE matrices.

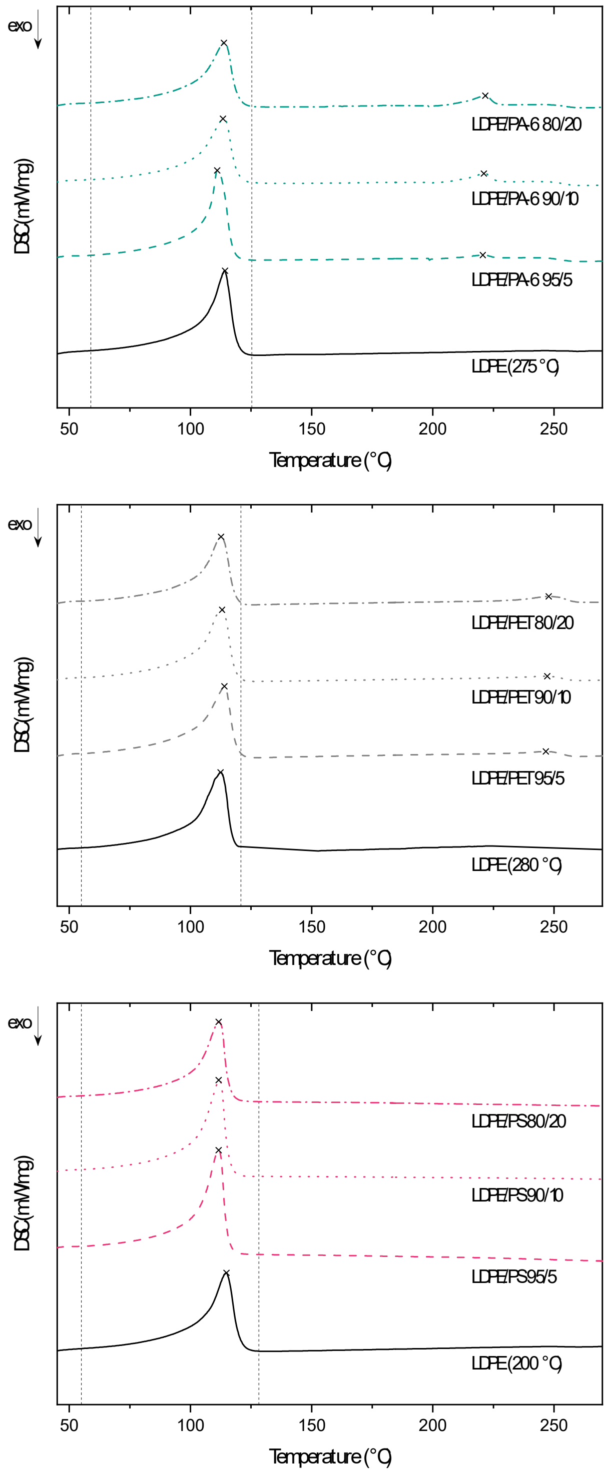

Figure S2: DSC signals of the NPO contaminated LDPE matrices.

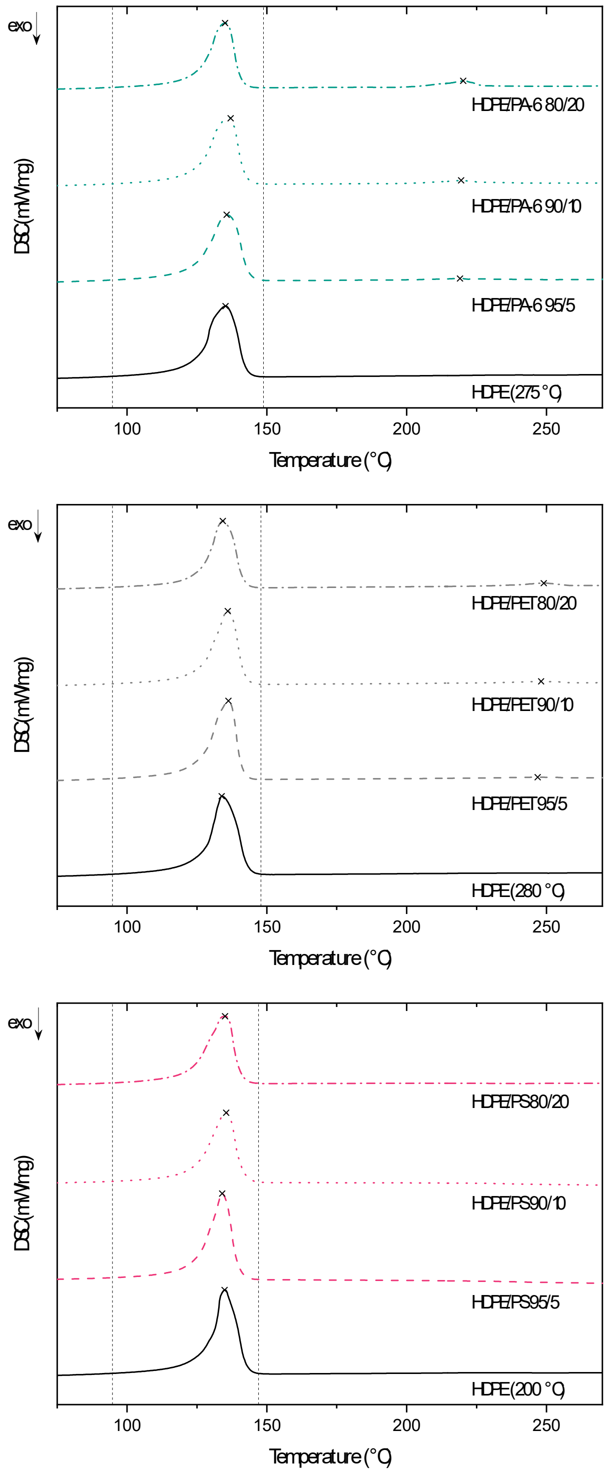

Figure S3: DSC signals of the NPO contaminated HDPE matrices.

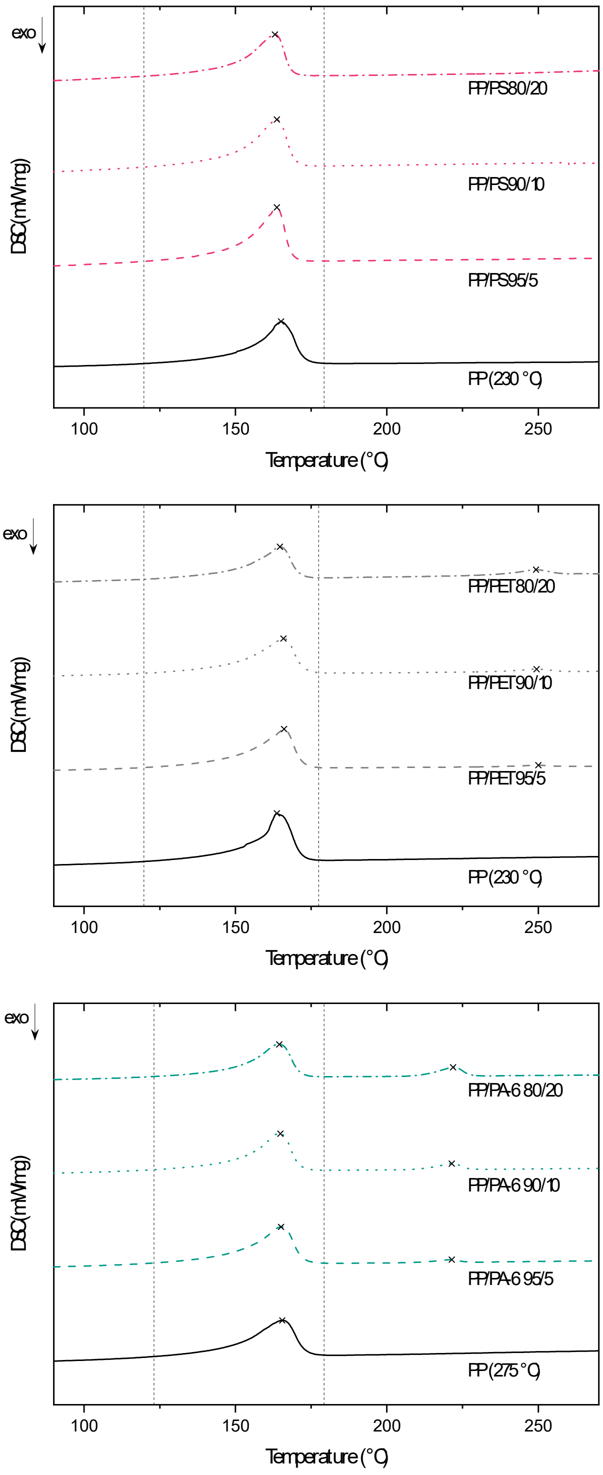

Figure S4: DSC signals of the NPO contaminated PP matrices.

2. Rotational rheology data of the used mono polymers

Rotational rheology on an Anton Paar MultiDrive MCR702 was performed to measure the complex viscosity, storage modulus and loss modulus of the mono materials used. The machine has a plate-plate geometry with a plate diameter of 25 mm and a gap of 1 mm. The measurements were performed in nitrogen atmosphere at 280 °C (highest processing temperature in the test series). All small amplitude oscillatory measurements were performed at strain 1% and at frequencies ranging from 100 s^{-1} to 1 s^{-1}. For all materials the relaxation time is lower than 0.01 s at 280 °C.

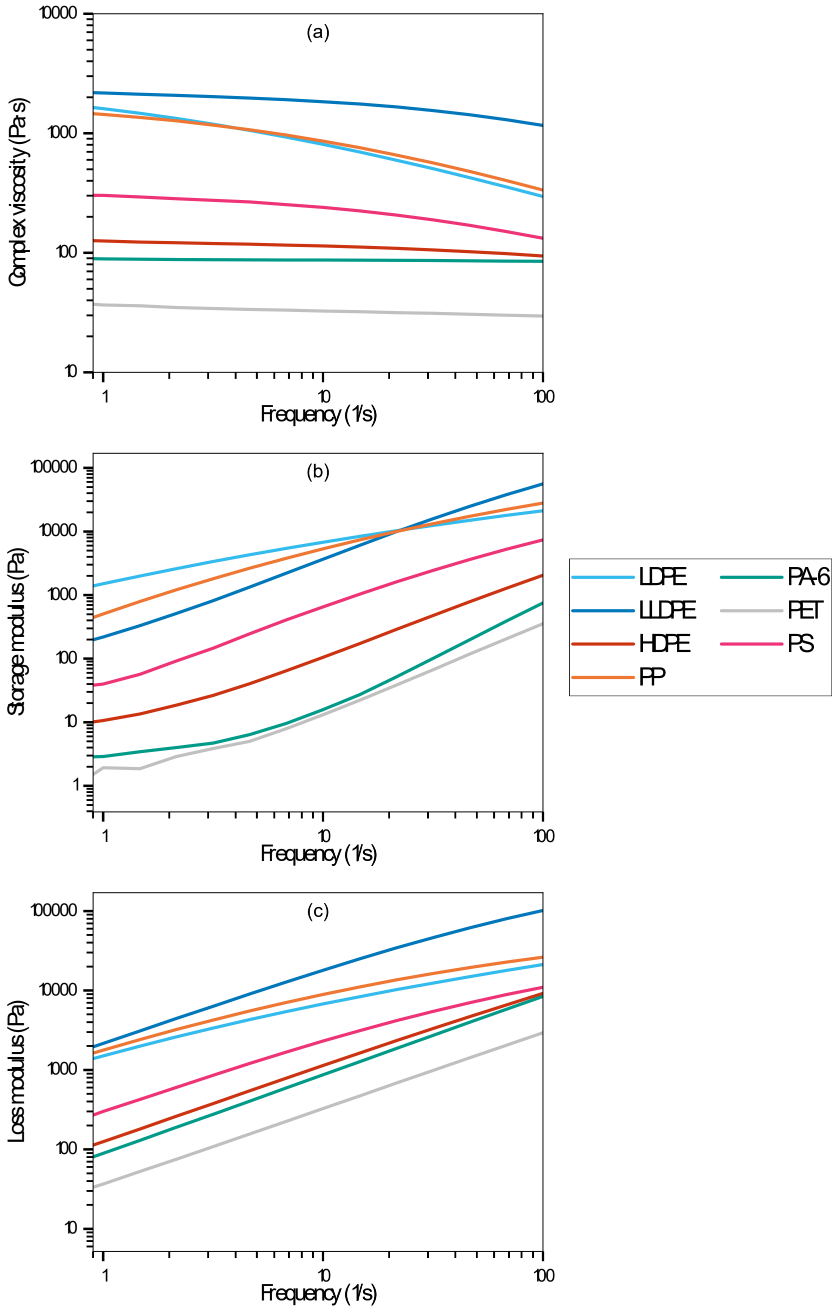

Figure S5: Rotational rheology results for the mono materials used: (a) complex viscosity, (b) Storage modulus and (c) Loss modulus.

3. Extra SEM images for the PA-6 and PS contaminated HDPE matrices.

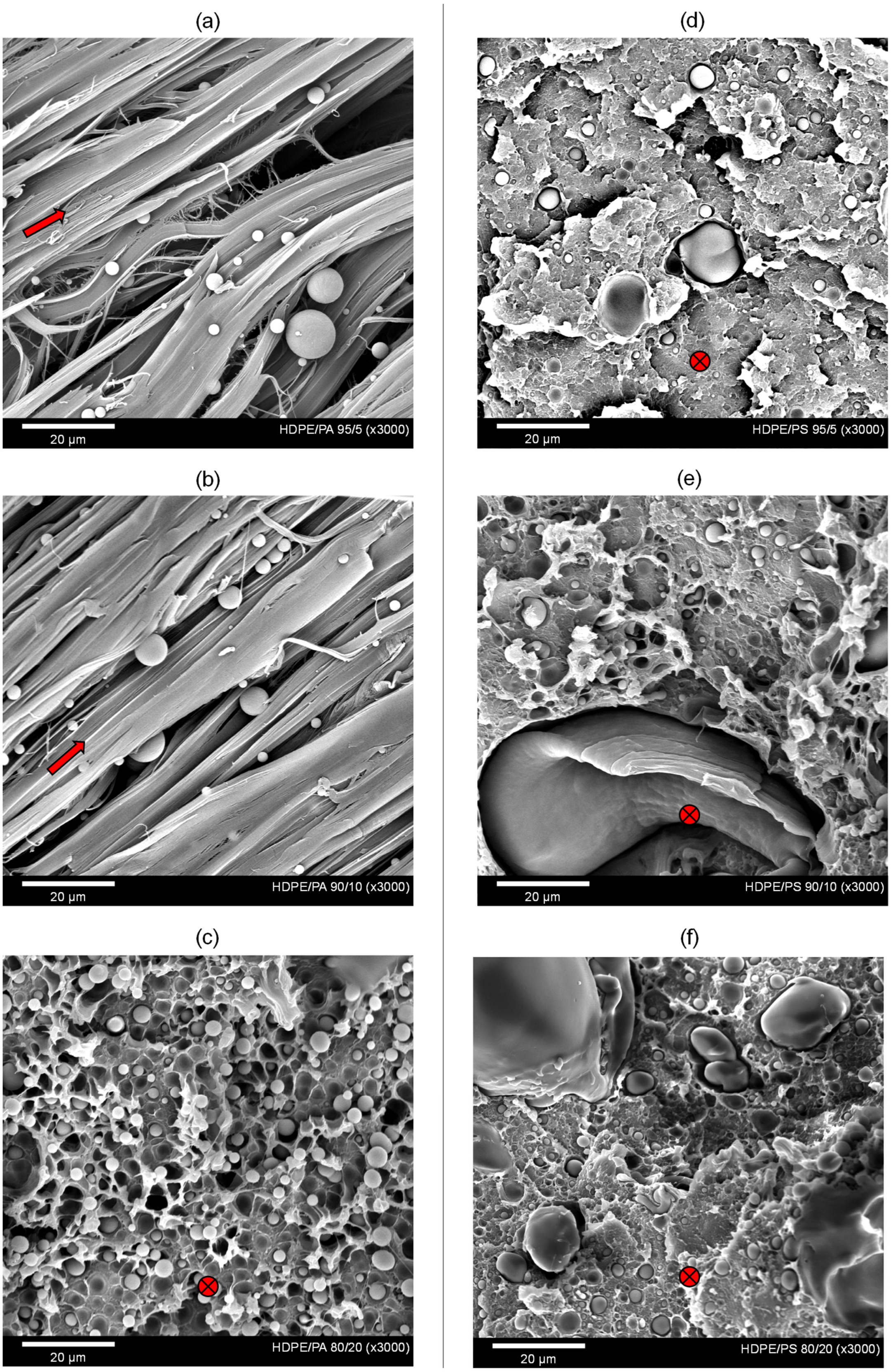

Figure S6: Scanning electron microscopy (SEM) images of the area of rupture from the tensile tested (deformed) binary blend specimens: (a) HDPE/PA 95/5, (b) HDPE/PA 90/10, (c) HDPE/PA 80/20, (d) HDPE/PS 95/5, (e) HDPE/PS 90/10 and (f) HDPE/PS 80/20. The red arrow indicates the polymer flow.

4. Mechanical properties of the investigated PO-NPO blends

Table S1: Tensile properties data of the different PO-NPO blend series.

Amount of LLDPE (wt%)	Amount of PA-6 (wt%)	E (MPa)	σ_y (MPa)	ε_y (%)	ε_b (%)
0	100	2260 ± 60	62.11 ± 0.36	4.09 ± 0.03	234.4 ± 101.8
5	95	1956 ± 118	52.94 ± 0.79	3.91 ± 0.12	142.6 ± 51.1
10	90	1883 ± 309	52.98 ± 1.06	3.77 ± 0.11	87.2 ± 17.1
20	80	1794 ± 25	44.94 ± 0.52	3.87 ± 0.09	73.1 ± 14.7
50	50	1057 ± 56	26.48 ± 0.66	5.30 ± 0.17	40.8 ± 2.2
80	20	254 ± 19	7.71 ± 0.32	2.63 ± 0.29	326.9 ± 11.8
90	10	156 ± 9	6.10 ± 0.15	3.07 ± 0.24	513.0 ± 32.7
95	5	131 ± 10	5.88 ± 0.08	3.48 ± 0.28	476.6 ± 39.4
100	0	109 ± 8	5.20 ± 0.17	3.51 ± 0.35	506.9 ± 17.3

Amount of LLDPE (wt%)	Amount of PET (wt%)	E (MPa)	σ_y (MPa)	ε_y (%)	ε_b (%)
0	100	2337 ± 218	49.39 ± 15.70	3.08 ± 1.60	2.9 ± 2.5
5	95	2140 ± 192	55.40 ± 0.98	4.07 ± 0.06	105.6 ± 77.9
10	90	2004 ± 74	47.45 ± 0.69	4.01 ± 0.08	162.3 ± 71.4
20	80	1747 ± 133	39.18 ± 1.98	3.98 ± 0.08	43.0 ± 10.7
50	50	874 ± 33	20.73 ± 0.90	4.66 ± 0.19	118.2 ± 21.9
80	20	228 ± 19	6.05 ± 0.25	2.37 ± 0.10	NB
90	10	190 ± 23	5.20 ± 0.29	2.39 ± 0.22	NB
95	5	151 ± 9	4.81 ± 0.12	2.67 ± 0.19	622.4 ± 26.5
100	0	120 ± 4	4.39 ± 0.11	2.96 ± 0.19	568.5 ± 7.2

Amount of LLDPE (wt%)	Amount of PS (wt%)	E (MPa)	σ_y (MPa)	ε_y (%)	ε_b (%)
0	100	3084 ± 42	52.52 ± 0.59	4.33 ± 0.17	5.2 ± 0.6
5	95	2991 ± 80	42.05 ± 3.68	2.30 ± 0.31	2.3 ± 0.3
10	90	2788 ± 105	39.08 ± 5.73	2.32 ± 0.54	2.3 ± 0.5
20	80	2546 ± 189	36.58 ± 1.41	2.29 ± 0.19	2.3 ± 0.2
50	50	1317 ± 75	29.23 ± 1.12	4.17 ± 0.35	4.2 ± 0.4
80	20	394 ± 39	8.36 ± 0.41	2.05 ± 0.12	444.3 ± 103.5
90	10	214 ± 10	6.10 ± 0.20	2.53 ± 0.05	583.0 ± 31.3
95	5	156 ± 10	5.46 ± 0.23	2.99 ± 0.14	480.8 ± 62.4
100	0	97 ± 3	5.34 ± 0.08	4.00 ± 0.19	532.8 ± 8.8

Amount of LDPE (wt%)	Amount of PA-6 (wt%)	E (MPa)	σ_y (MPa)	ε_y (%)	ε_b (%)
0	100	2260 ± 60	62.11 ± 0.36	4.09 ± 0.03	234.4 ± 101.8
5	95	2230 ± 81	56.41 ± 1.36	3.87 ± 0.39	80.4 ± 34.2
10	90	2030 ± 39	52.02 ± 0.85	4.26 ± 0.09	65.6 ± 10.0
20	80	1733 ± 48	44.07 ± 0.69	5.75 ± 0.45	41.5 ± 12.9
50	50	990 ± 184	24.67 ± 3.01	4.89 ± 0.42	13.1 ± 4.7
80	20	372 ± 33	10.02 ± 0.49	2.43 ± 0.11	25.5 ± 6.9
90	10	236 ± 18	8.47 ± 0.23	3.1 ± 0.17	111.6 ± 6.8
95	5	186 ± 7	7.60 ± 0.12	3.41 ± 0.10	112.1 ± 5.4
100	0	154 ± 5	7.08 ± 0.05	3.73 ± 0.12	119.5 ± 2.0

Amount of LDPE (wt%)	Amount of PET (wt%)	E (MPa)	σ_y (MPa)	ε_y (%)	ε_b (%)
0	100	2337 ± 218	49.39 ± 15.70	3.08 ± 1.60	2.9 ± 2.5
5	95	2095 ± 102	52.63 ± 1.12	4.32 ± 0.06	32.9 ± 11.0
10	90	1940 ± 147	45.42 ± 4.10	3.42 ± 0.10	10.5 ± 6.2
20	80	1542 ± 84	35.84 ± 1.74	3.28 ± 0.07	13.7 ± 3.8
50	50	858 ± 18	20.33 ± 0.42	4.95 ± 0.16	5.4 ± 0.4
80	20	301 ± 32	9.51 ± 0.56	3.21 ± 0.36	48.9 ± 14.3
90	10	249 ± 39	7.07 ± 0.68	2.99 ± 0.65	82.9 ± 7.9
95	5	206 ± 33	6.37 ± 0.76	3.26 ± 0.94	97.9 ± 11.0
100	0	173 ± 37	6.50 ± 0.56	4.01 ± 0.88	132.1 ± 4.7

Amount of LDPE (wt%)	Amount of PS (wt%)	E (MPa)	σ_y (MPa)	ε_y (%)	ε_b (%)
0	100	3084 ± 42	52.52 ± 0.59	4.33 ± 0.17	5.2 ± 0.6
5	95	2928 ± 37	47.82 ± 0.60	2.69 ± 0.18	2.7 ± 0.2
10	90	2604 ± 70	43.23 ± 0.77	2.42 ± 0.14	2.4 ± 0.1
20	80	2268 ± 86	36.78 ± 0.89	2.16 ± 0.09	2.2 ± 0.1
50	50	1331 ± 95	26.74 ± 1.13	2.98 ± 0.21	3.0 ± 0.2
80	20	605 ± 62	12.32 ± 0.79	2.07 ± 0.09	15.5 ± 5.9
90	10	351 ± 20	8.54 ± 0.29	2.34 ± 0.13	50.6 ± 6.0
95	5	251 ± 15	7.49 ± 0.20	2.77 ± 0.17	69.3 ± 6.8
100	0	165 ± 9	5.45 ± 0.27	3.20 ± 0.28	97.0 ± 3.8

Amount of HDPE (wt%)	Amount of PA-6 (wt%)	E (MPa)	σ_y (MPa)	ε_y (%)	ε_b (%)
0	100	2260 ± 60	62.11 ± 0.36	4.09 ± 0.03	234.4 ± 101.8
5	95	2052 ± 180	53.08 ± 1.97	3.64 ± 0.07	318.4 ± 71.3
10	90	2095 ± 60	51.28 ± 0.74	3.52 ± 0.09	134.6 ± 35.8
20	80	2001 ± 45	44.80 ± 0.66	3.46 ± 0.09	50.3 ± 7.5
50	50	1394 ± 39	24.46 ± 0.32	3.61 ± 0.07	6.6 ± 0.5
80	20	1043 ± 21	22.34 ± 0.37	6.71 ± 0.34	13.8 ± 1.1
90	10	921 ± 34	23.16 ± 0.30	8.41 ± 0.30	110.2 ± 48.8
95	5	877 ± 39	23.08 ± 1.04	9.21 ± 0.25	201.8 ± 69.2
100	0	893 ± 61	24.99 ± 0.48	10.30 ± 0.21	206.4 ± 31.5

Amount of HDPE (wt%)	Amount of PET (wt%)	E (MPa)	σ_y (MPa)	ε_y (%)	ε_b (%)
0	100	2337 ± 218	49.39 ± 15.7	3.08 ± 1.60	2.9 ± 2.5
5	95	2061 ± 74	54.89 ± 1.06	5.56 ± 0.08	364.2 ± 129.2
10	90	2501 ± 429	54.26 ± 1.25	5.44 ± 0.18	13.6 ± 3.8
20	80	1986 ± 126	45.02 ± 1.15	4.32 ± 0.12	6.0 ± 0.8
50	50	1587 ± 66	21.37 ± 1.29	2.32 ± 0.50	2.4 ± 0.5
80	20	1115 ± 20	23.86 ± 0.18	4.77 ± 0.07	9.8 ± 1.6
90	10	1011 ± 18	24.10 ± 0.20	6.83 ± 0.28	121.5 ± 39.3
95	5	913 ± 38	23.75 ± 0.26	8.43 ± 0.14	364.5 ± 129.3
100	0	868 ± 19	24.36 ± 0.16	10.29 ± 0.08	207.8 ± 33.4

Amount of HDPE (wt%)	Amount of PS (wt%)	E (MPa)	σ_y (MPa)	ε_y (%)	ε_b (%)
0	100	3084 ± 42	52.52 ± 0.59	4.33 ± 0.17	5.2 ± 0.6
5	95	3017 ± 38	41.48 ± 5.50	2.25 ± 0.41	2.4 ± 0.2
10	90	2858 ± 36	35.67 ± 1.70	1.98 ± 0.15	2.0 ± 0.1
20	80	2610 ± 42	31.19 ± 2.21	1.83 ± 0.17	1.8 ± 0.2
50	50	1781 ± 58	20.46 ± 0.56	1.76 ± 0.08	1.8 ± 0.1
80	20	1171 ± 48	20.34 ± 0.35	3.86 ± 0.42	4.3 ± 0.8
90	10	1030 ± 50	21.80 ± 0.37	6.57 ± 0.73	7.5 ± 1.2
95	5	1024 ± 37	22.54 ± 0.40	7.73 ± 1.24	9.5 ± 1.9
100	0	984 ± 14	24.19 ± 0.27	10.29 ± 0.71	219.7 ± 13.1

Amount of PP (wt%)	Amount of PA-6 (wt%)	E (MPa)	σ_y (MPa)	ε_y (%)	ε_b (%)
0	100	2260 ± 60	62.11 ± 0.36	4.09 ± 0.03	234.4 ± 101.8
5	95	2363 ± 49	55.11 ± 0.65	3.93 ± 0.05	195.2 ± 67.8
10	90	2405 ± 21	52.83 ± 0.51	4.25 ± 0.07	61.9 ± 7.2
20	80	2310 ± 19	45.54 ± 0.99	4.53 ± 0.11	36.3 ± 6.8
50	50	1891 ± 23	33.01 ± 0.40	4.60 ± 0.12	15.3 ± 2.5
80	20	1739 ± 24	29.82 ± 0.40	5.24 ± 0.09	11.9 ± 1.7
90	10	1742 ± 22	32.77 ± 0.27	6.13 ± 0.13	17.5 ± 1.9
95	5	1849 ± 18	35.19 ± 0.22	6.43 ± 0.09	15.2 ± 3.8
100	0	1672 ± 30	36.88 ± 0.25	7.90 ± 0.10	45.3 ± 8.6

Amount of PP (wt%)	Amount of PET (wt%)	E (MPa)			σ_y (MPa)			ε_y (%)			ε_b (%)		
0	100	2337	±	218	49.39	±	15.7	3.08	±	1.60	2.9	±	2.5
5	95	2403	±	153	55.60	±	1.33	3.95	±	0.62	7.2	±	1.0
10	90	2395	±	129	47.38	±	0.76	2.99	±	0.41	12.8	±	2.4
20	80	2246	±	120	41.98	±	1.38	2.80	±	0.03	7.0	±	1.8
50	50	1877	±	56	33.96	±	0.60	3.45	±	0.08	6.7	±	1.2
80	20	1885	±	44	31.62	±	0.58	4.62	±	0.17	6.0	±	0.8
90	10	1827	±	30	32.57	±	0.39	5.47	±	0.28	7.1	±	0.4
95	5	1831	±	30	34.00	±	0.58	6.38	±	0.55	8.7	±	0.9
100	0	1745	±	32	36.04	±	0.14	7.59	±	0.07	52.9	±	10.2

Amount of PP (wt%)	Amount of PS (wt%)	E (MPa)			σ_y (MPa)			ε_y (%)			ε_b (%)		
0	100	3202	±	37	49.67	±	0.30	3.62	±	0.07	4.6	±	0.4
5	95	3090	±	44	36.22	±	6.73	1.58	±	0.46	1.6	±	0.5
10	90	2978	±	30	39.30	±	5.17	1.77	±	0.42	2.0	±	0.1
20	80	2958	±	20	42.79	±	1.08	1.95	±	0.11	2.0	±	0.1
50	50	2361	±	46	38.68	±	0.55	2.65	±	0.19	2.7	±	0.2
80	20	1867	±	33	37.39	±	0.56	5.26	±	0.16	6.8	±	1.0
90	10	1745	±	19	36.16	±	0.17	5.97	±	0.12	17.3	±	6.4
95	5	1718	±	42	36.12	±	0.26	6.95	±	0.14	100.9	±	30.0
100	0	1706	±	29	36.81	±	0.29	7.87	±	0.07	141.8	±	19.5
